# Genomic exploration of *Sesuvium sesuvioides*: comparative study and phylogenetic analysis within the order Caryophyllales from Cholistan desert, Pakistan

**DOI:** 10.1186/s12870-023-04670-5

**Published:** 2023-12-20

**Authors:** Nida Javaid, Musarrat Ramzan, Shagufta Jabeen, Muhammad Nadeem Shah, Subhan Danish, Abdurahman Hajinur Hirad

**Affiliations:** 1 Department of Botany, Faculty of Chemical and Biological Sciences, The Islamia University Bahawalpur, Bahawalpur, Punjab Pakistan; 2 Government Associate College for Women Ahmedpur East, Bahawalpur, Punjab Pakistan; 3 Department of Agriculture, Government College University Lahore, Lahore, Punjab Pakistan; 4 North Florida Research and Education Center, University of Florida, 155 Research Road, Quincy, Florida USA; 5 Department of Soil Science, Faculty of Agricultural Sciences and Technology, Bahauddin Zakariya University, Multan, Punjab Pakistan; 6 Department of Botany and Microbiology, College of Science, King Saud University, P. O. Box.2455, Riyadh, 11451 Saudi Arabia

**Keywords:** Aizoaceae, Caryophyllales, *Sesuvium sesuvioides*, *Sesuvium portulacastrum*, *Tetragonia tetragonoides*

## Abstract

**Background:**

The Aizoaceae family’s *Sesuvium sesuvioides* (Fenzl) Verdc is a medicinal species of the Cholistan desert, Pakistan. The purpose of this study was to determine the genomic features and phylogenetic position of the *Sesuvium* genus in the Aizoaceae family. We used the Illumina HiSeq2500 and paired-end sequencing to publish the complete chloroplast sequence of *S. sesuvioides*.

**Results:**

The 155,849 bp length cp genome sequence of *S. sesuvioides* has a 36.8% GC content. The Leucine codon has the greatest codon use (10.6%), 81 simple sequence repetitions of 19 kinds, and 79 oligonucleotide repeats. We investigated the phylogeny of the order Caryophyllales’ 27 species from 23 families and 25 distinct genera. The maximum likelihood tree indicated *Sesuvium* as a monophyletic genus, and sister to *Tetragonia*. A comparison of *S. sesuvioides*, with *Sesuvium portulacastrum*, *Mesembryanthemum crystallinum*, *Mesembryanthemum cordifolium*, and *Tetragonia tetragonoides* was performed using the NCBI platform. In the comparative investigation of genomes, all five genera revealed comparable cp genome structure, gene number and composition. All five species lacked the *rps15* gene and the *rpl2* intron. In most comparisons with *S. sesuvioides*, transition substitutions (Ts) were more frequent than transversion substitutions (Tv), producing Ts/Tv ratios larger than one, and the Ka/Ks ratio was lower than one. We determined ten highly polymorphic regions, comprising *rpl22*, *rpl32-trnL-UAG*, *trnD-GUC-trnY-GUA*, *trnE-UUC-trnT-GGU*, *trnK-UUU-rps16*, *trnM-CAU-atpE*, *trnH-GUG-psbA*, *psaJ-rpl33*, *rps4-trnT-UGU*, and *trnF-GAA-ndhJ.*

**Conclusion:**

The whole *S. sesuvioides* chloroplast will be examined as a resource for in-depth taxonomic research of the genus when more *Sesuvium* and Aizoaceae species are sequenced in the future. The chloroplast genomes of the Aizoaceae family are well preserved, with little alterations, indicating the family’s monophyletic origin. This study’s highly polymorphic regions could be utilized to build realistic and low-cost molecular markers for resolving taxonomic discrepancies, new species identification, and finding evolutionary links among Aizoaceae species. To properly comprehend the evolution of the Aizoaceae family, further species need to be sequenced.

**Supplementary Information:**

The online version contains supplementary material available at 10.1186/s12870-023-04670-5.

## Introduction

The Caryophyllales order is made up of 37 families, 749 genera, and around 11,600 species [1, 2]. The clade is famous not only for its rich diversity and wide ecological and geographic distribution but also for a variety of distinct morphological and eco-physiological adaptations [3]. Many Caryophyllales species are well-known for their drought resistance, but the clade also includes species with high adaptations to cold, heavy metal hyper-accumulation, salt tolerance, carnivory, CAM and C4 metabolism, and succulence [3–6]. The majority of these characteristics are known to have evolved at different times throughout the group, making Caryophyllales an important natural laboratory for studying evolutionary traits in angiosperms [3]. Aizoaceae is the largest succulent family of the Caryophyllales order with five sub-families, 143 genera, and more than 2300 species that are typically located in tropical and subtropical climates, especially along the shore or in arid areas [7]. The five Aizoaceae sub-families are Tetragonioideae, Mesembryanthemoideae, Sesuvioideae, Ruschioideae and Aizooideae [4, 8]. The subfamily Sesuvioideae is reported to have two tribes (Sesuvieae and Anisostigmateae) and five genera (*Zaleya*, *Sesuvium*, *Trianthema*, *Cypselea*, and *Tribulocarpus*) [4, 6]. Molecular and morphological phylogenetic studies demonstrate that Sesuvioideae is closely associated with the other four Aizoaceae subfamilies [6]. Sesuvioideae and its sibling group separated at the beginning of the Miocene period [9] [10];. During the late Miocene, the sister clades, especially the Ruschioideae, produced about 1770 species, whereas the Sesuvioideae retained a low number of species [4, 9, 10]. Molecular phylogenetic studies also demonstrated that Mesembryanthemoideae and Ruschioideae species are monophyletic [8, 9].

**Fig. 1:**
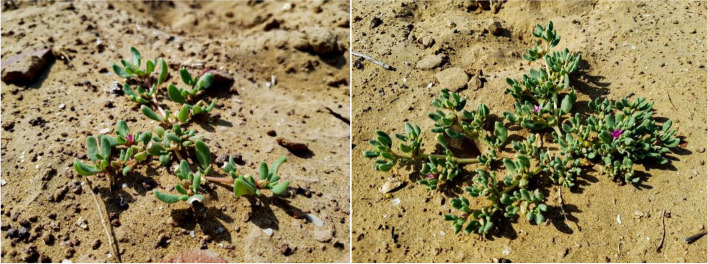
The *Sesuvium sesuvioides* (*Barri Ulwaiti*) growing in the Cholistan desert

One of the most widespread genera in the Sesuvioideae subfamily, *Sesuvium* L. is found all over the world in diverse subtropical and tropical climates [11, 12]. According to previous morphological and molecular phylogenetic investigations, there were fourteen to seventeen accepted species of the *Sesuvium* genus [13]. Due to the absence of well-defined species boundaries, *Sesuvium* is one of Aizoaceae’s more challenging genera to categorize [5]. Barri Ulwaiti is the local name for *Sesuvium sesuvioides* (Fenzl) Verdc. (Aizoaceae). *S. sesuvioides* is a short-lived perennial herb and one of the succulent halophytes found mostly in salty areas and inter-dunal clayey plains of Pakistan’s Cholistan desert [14] shown in Fig. 1. It has 2n = 16 chromosomes [15]. *S. sesuvioides* is a herbal medicine utilized by indigenous practitioners in the Cholistan desert for folkloric cures such as arthritis, gout, epistaxis, bleeding, smallpox, chickenpox, cold, flu, haemorrhage, thyroid malfunction, inflammation, fever, ulcer, measles, and nasal bleeding [16–18]. Aqueous extracts of this plant contained phenolic compounds, flavonoids, glycosides, coumarin, terpenes, saponins, triglycerides, and carbohydrates [19]. Recent research has demonstrated its anti-inflammatory, analgesic, and antipyretic effects [19].

Chloroplasts in higher plants serve as metabolic hubs for photosynthesis, which merely keeps life on Earth alive [20]. Chloroplast (cp.) genomes are significant and relevant data sources for evolutionary biology, and they have been utilized extensively in plant phylogenetic investigations [21]. The cp genome has significantly conserved gene content and genome order [22]. Due to its modest size, the chloroplast genome was the inaugural plant genome to be thoroughly sequenced [23]. Furthermore, the cp genome has fewer nucleotide alterations and genome sequence reorganizations than the nuclear genome [24, 25] making it a great tool for figuring out how genomes evolve and how phylogenetics relations work in complicated angiosperm families [21, 26, 27]. The phylogenetic investigations of various plant families have benefited tremendously from the use of chloroplast genomes, which have also made it easier to understand the evolutionary connections between various phylogenetic clades [21]. Our perception of plant science and heterogeneity has been expanded by the whole chloroplast genome sequencing [28].

Even though Aizoaceae species have received significant research attention in terms of evolutionary studies [6, 9, 29, 30], there is very little data for chloroplast genomes on NCBI, and information for phylogenetic connections within major subfamilies is currently not sufficient [9]. Only four cp genomes of Aizoaceae species (*Sesuvium portulacastrum*, *Mesembryanthemum crystallinum*, *Mesembryanthemum cordifolium*, and *Tetragonia tetragonoides*) have been completely sequenced available on NCBI [1]. Hassan et al., (2005) previously explored the phylogeny of Sesuvioideae (Aizoaceae) based on nuclear DNA, but the location of the genus *Sesuvium* remained unknown [29]. To resolve phylogenetic difficulties, more chloroplast genomes from the genus *Sesuvium* (Aizoaceae family) must be sequenced and analyzed. Understanding the evolutionary link between *S. sesuvioides* and other Aizoaceae plants can help in the sequencing and identification of other members of the family. This study aimed to fill some knowledge gaps by (1) gaining insights into the structure of the *S. sesuvioides* plastid genome and performing comparative analysis to explore mutations within the Aizoaceae family, (2) reconstructing the phylogenetic tree of the Caryophyllales order to resolve phylogenetic issues in both the genus and the family, and (3) determining highly polymorphic loci for the creation of reliable, significant, and affordable species detection markers. This study will aid in adding substantial molecular and phylogenetic data to the Aizoaceae family to accommodate species detection in the future.

## Materials and methods

### DNA extraction and chloroplast genome sequencing

Plants of *S. sesuvioides* were collected from Pakistan’s Lesser Cholistan desert (28.7719699, 71.3346211) and the regulatory verification procedure was conducted at the Cholistan Institute of Desert Studies (CIDS) of Islamia University Bahawalpur. The DNA was extracted from fresh and young leaves using the SDS-based DNA extraction method [31]. The quantity and quality of isolated DNA were assessed using nanodrop and 1% agarose gel electrophoresis. A whole genome shotgun was created at the Beijing Institute of Genomics (BIG) in Beijing, China, using an Illumina Hiseq2500 with a Paired-end database containing 150 bp.

### Annotation of genes and assembly of the chloroplast genome

FastQC analysis was used to assess the quality of the raw readings data [32]. We used NOVOPlasty to extract the chloroplast genome from whole genome sequence (WGS) data and reconstructed the full chloroplast genome [33]. By evaluating the sequence scaffolding, the borders of the LSC, SSC, and IR regions were observed. GeSeq [34] and CpGAVAS with standard settings were used to annotate the cp genome sequence [35]. For further verification of annotations, the *S. sesuvioides* genome was pairwise aligned with other genomes of the Aizoaceae family, *S. portulacastrum* (MK330004), *M. crystallinum* (KM016695), *M. cordifolium* (MK397873), and *T. tetragonoides* (MF975369) by MAFFT alignment (Multiple Alignment with Fast Fourier Transform) [36] in Geneious Prime 2021.1.1 [37]. The tRNAscan-SE 1.23 programme was employed to validate the tRNA genes [38]. By mapping sequencing short sequences to their corresponding de novo assembled cp genomes using BWA software [39], the average sequencing coverage depth for the assembled *S. sesuvioides* genome was obtained and visualized in Tablet [40]. OGDraw v1.2 [40] was used to build the circular map of the cp genome. The cp genome of *S. sesuvioides* was submitted to GenBank and assigned the accession number MW539047. The raw data acquired in this investigation was uploaded to Sequence Read Archive (SRA) under project number PRJNA660981.

### Determination of amino acid frequency, and codon usage

MEGA-X [41] was used to examine Relative Synonymous Codon Usage (RSCU) in *S. sesuvioides* protein-coding sequences, whereas Geneious Prime 2021.1.1 was used to examine amino acid frequency [37].

### Detection of simple sequence repeats (SSRs) and oligonucleotide repeats

The Perl script MIcroSAtellite Identification Tools (MISA) [42] was used to detect SSRs, with minimum repetition counts of ten for mono-, five for di-, four for tri-, three tetra-, three Penta-, and three for hexanucleotides. The REPuter programme [43] was used to find forward (F), reverse (R), complementary (C), and palindromic (P) oligonucleotide repeats with an edit distance of two, a minimum repeat size of 10 bp, and a maximum computed repeat of 100.

### Phylogenetic analysis of Caryophyllales

The cp genomes of 26 species of order Caryophyllales from 23 families were used to construct phylogenetic connections (Table S5). *Asclepias nivea* and *Asclepias syriaca* from the Apocynaceae family were chosen as an outgroup. The phylogenetic tree contains 29 species in total (1 *S. sesuvioides* plus 28 NCBI species). The selected species were downloaded from NCBI (National Center for Biotechnology Information), protein-coding sequences from each species were extracted, and the sequences were concatenated in Geneious Prime 2021.1.1. To align these protein-coding sequences, MAFFT was employed. The best-fit model GTR + F + R6 according to AIC (Akaike information criterion) was used to build the phylogenetic tree [44]. The maximum likelihood tree was generated online in Galaxy using IQ-TREE [45], and Ultrafast bootstrap settings with 1000 bootstrap replications [46]. To complete the tree display, we utilized the iTOL (interactive tree of life) software [47].

### Species selected for basic comparison with *S. Sesuvioides*

Based on the results of phylogenetic studies, the complete cp genomes of four Aizoaceae species including *S. portulacastrum*, *M. crystallinum*, *M. cordifolium*, and *T. tetragonoides* were compared to that of *S. sesuvioides*. The Geneious Prime 2021.1.1 was used to analyze the cp genomes for the basic comparison. The Mafft alignment was used to make the multiple alignments of the selected species, to detect the arrangement and show the comparison between these cp genomes.

### IR contraction and expansion

The junctions of cp genomes of five Aizoaceae species, including *S. sesuvioides*, *S. portulacastrum*, *M. crystallinum*, *M. cordifolium*, and *T. tetragonoides*, were compared. With the use of IRScope, the expansion and contraction of IRs areas at the intersections of the four major cp genome components (LSC/IRb/SSC/IRa) were studied [48].

### Estimation of synonymous (Ks) and non-synonymous (Ka) substitution rates

We analyzed synonymous (Ks), non-synonymous substitutions (Ka), and Ka/Ks values by making pair-wise alignments of protein-coding sequences of *S. sesuvioides* cp genome with the other four species of Aizoaceae. To do this, we extracted the protein-coding sequences for each genome and performed the pairwise alignment using Geneious Prime 2021.1.1. Pairwise alignments for each analysis were carried out using *S. sesuvioides* as the reference member. DnaSP was utilized to assess the pairwise alignment and to identify Ka, and Ks substitutions [49].

### Single nucleotide polymorphisms (SNPs) and InDels mutations

IR, SSC, and LSC areas of the *S. sesuvioides* chloroplast genome were pair-wise aligned with corresponding regions of the other four Aizoaceae species cp genomes chosen for comparative study using MAFFT alignment implemented in Geneious Prime 2021.1.1. The reference genome for this investigation was the *S. sesuvioides* cp genome. The Geneious Prime 2021.1.1 was used to calculate the number, spatial positions, and kind of SNPs (transition and transversion). The pairwise aligned cp genomes were used to find InDels mutations using DnaSP [49]. For each site, it also calculated the alignment length, no. of inDels, k(i) inDel diversity, inDel average length, and Pi(i) inDel diversity per site.

### Determination of nucleotide diversity and high polymorphism loci

Nucleotide diversity (π) was calculated in 130 common regions of *S. sesuvioides*, *S. portulacastrum*, *M. crystallinum*, *M. cordifolium*, and *T. tetragonoides*. We made 130 multiple alignments in MAFFT from 650 regions extracted from the five Aizoaceae species (60 CDS regions, 14 intronic locations and 50 IGS sequences). Nucleotide diversity (π) was calculated using DnaSP [49]. To observe highly polymorphic regions between the species chosen for comparative study, ten loci with greater nucleotide diversity were selected.

## Results

### Chloroplast genome structure of *Sesuvium sesuvioides*

**Fig. 2:**
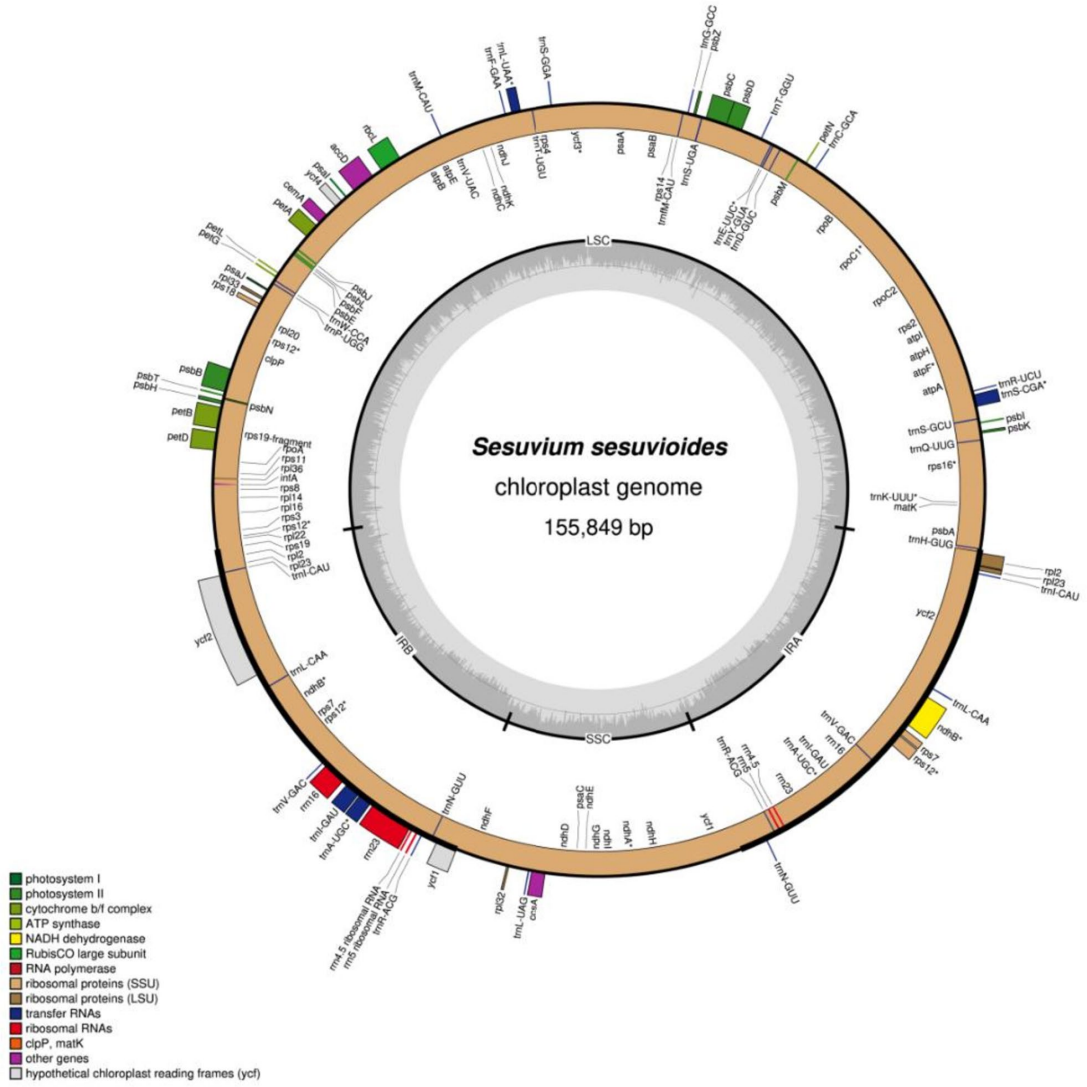
The cp genomic map of *S. sesuvioides*. Genes are translated in two directions: clockwise for those on the circle’s rim and anticlockwise for those within. Protein-coding genes are distinguished by their shade based on their function. In the inner circle, the AT and GC components of the genome are depicted as light grey and dark grey, respectively. Inverted Repeats are denoted by the letters IRb and IRa, whereas Single-copy regions are denoted by the letters SSC and LSC

The raw data for *S. sesuvioides* from the Illumina HiSeq2500 paired-end sequencing with 150 bp reads was 10.9 GB. The de novo assembled *S. sesuvioides* cp genome had an average coverage depth of 870. The *S. sesuvioides* cp genome (155,849 bp) is composed of the SSC region (18,736 bp), the LSC region (89,619 bp), and two inverted repeats (25,947 bp). It has 36.8% GC overall, with IRs having 42.8% greater GC than the LSC (34.6%) and SSC (30.2%). The 132 genes found in the chloroplast genome of *S. sesuvioides* comprise 37 tRNA, eight rRNA, and 87 CDS genes. In the inverted repeat regions, 19 genes are duplicated (Table 1). The genome of *S. sesuvioides* cp comprises 21 genes with introns, including 13 CDS genes and 8 tRNA genes. There are 18 genes with one intron and three with two introns (Table 2; Fig. 2), including *ycf3*, *rps12*, and *clpP.* The *rps12* gene showed evidence of trans-splicing. The *S. sesuvioides* cp genome lacked the *rpl2* intron and the *rps15* genes. The *ycf1* gene began in the inverted repeats and ended in the SSC domain, with a pseudo copy found in the IRB region. Similarly, at the IRB/LSC junction, the gene *rps19* has a functional copy of 279 bp and a pseudo copy of 150 bp. The *S. sesuvioides* cp genome is available on this NCBI link: https://www.ncbi.nlm.nih.gov/nuccore/MW539047.1.

**Table 1:** The full *S. sesuvioides* cp genome’s comprehensive properties

Category	Items	Descriptions
**Construction of cp genome**	LSC region (bp)	85,619
	IRA region (bp)	25,747
	SSC region (bp)	18,736
	IRB region (bp)	25,747
	Genome Size (bp)	155,849
**Gene content**	Total genes	132
	Protein-coding genes	87
	tRNAs	37
	rRNAs	8
	Two copy genes	19 (8 CDS, 7 tRNA, 4 rRNA)
	Genes in the LSC region	84 (*rps12* repeated)
	Genes on IRA region	19
	Genes in SSC region	12 (10 CDS, 1 tRNA)
	Genes on IRB region	19
	Gene total length (bp)	112,089
	Average of genes length (bp)	849
	Gene length/Genome (%)	0.72
	Genes with pseudo copies	*rps19*, *ycf1*
**GC content**	GC content of LSC region (%)	34.6
	GC content of IRA region (%)	42.8
	GC content of SSC region (%)	30.2
	GC content of IRB region (%)	42.8
	Overall GC content (%)	36.8
**Intron containing genes**	Total intron-containing genes	21
	ICGs Protein coding (CDS)	13
	ICGs in tRNA	8
	ICGs in rRNA	0
	1 Intron-containing Genes	18
	2 Intron-containing Genes	*3* (*ycf3*, *clpP*, *rps12*)

**Table 2:** Genes containing introns and their length in *S. sesuvioides*

Genes	Strand	Gene length		Length	Exon I	Intron I	Exon II	Intron II	Exon III
		Start	End						
*clpP*	reverse	71,326	73,426	591	71	896	292	614	228
*ycf3*	reverse	43,259	45,301	507	126	777	228	759	153
*trnK-UUU*	reverse	1708	4283	72	37	2504	35		
*trnI-GAU*	forward	103,144	104,151	72	37	936	35		
*trnI-GAU*	reverse	137,318	138,325	72	37	936	35		
*trnA-UGC*	forward	104,222	105,114	73	38	820	35		
*trnA-UGC*	reverse	136,355	137,247	73	38	820	35		
*trnS-CGA*	forward	9211	10,009	91	31	708	60		
*trnV-UAC*	reverse	52,345	53,014	73	38	597	35		
*trnL-UAA*	forward	47,882	48,491	87	37	523	50		
*rpoC1*	reverse	20,856	23,713	2043	432	815	1611		
*ndhA*	reverse	121,771	123,975	1092	552	1113	540		
*ndhB*	reverse	95,692	97,892	1533	777	668	756		
*ndhB*	forward	143,577	145,777	1533	777	668	756		
*petB*	forward	76,436	77,837	648	6	754	642		
*rpl16*	reverse	82,634	84,029	411	9	985	402		
*atpF*	reverse	11,975	13,278	555	144	749	411		
*petD*	forward	78,041	79,232	483	8	709	475		
*rps16*	reverse	5084	6186	243	41	860	202		
*rps12*	mixed	71,038	142,740	372	114		231	547	27
*rps12*	reverse	71,038	99,533	372	114		231	547	27

### Amino acid frequencies and RSCU values

**Fig. 3:**
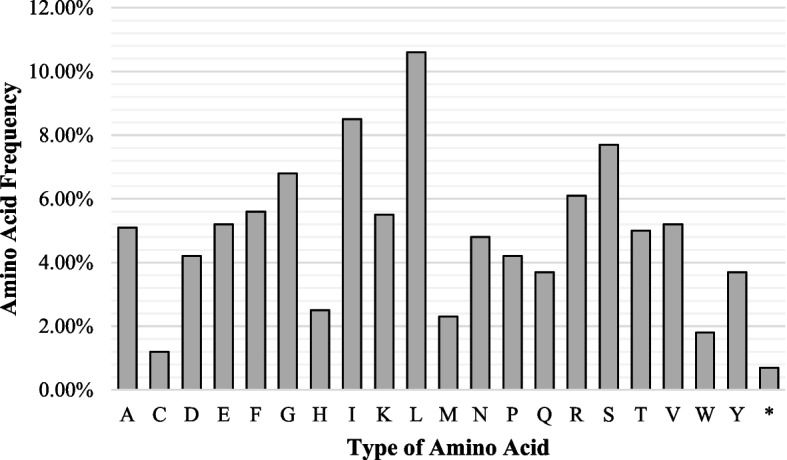
Comparison of amino acid frequency for *S. sesuvioides*

The *S. sesuvioides* contain a coding sequence of 80,376 bp and 51,949 codons. Leucine (11%) was the most prevalent amino acid in the *S. sesuvioides* cp genome, followed by isoleucine (9%), while cysteine (1%) was the least abundant amino acid (see Fig. 3). We discovered RSCU values for 64 codons, 31 of which were with RSCU values less than one. The RSCU values ranged from 0.44 to 1.81. The AGA codon, which encodes Arginine, had the highest usage bias (1.81), whereas the CGC codon, which also encodes Arginine, had the lowest (0.44). In the *S. sesuvioides* plastid genome, 12 codons exhibited the lowest preference (1.0 < RSCU< 1.2), two with no preference (RSCU = 1) 6 demonstrated modest preference (1.2 < RSCU< 1.3), and 13 exhibited intense preference (RSCU> 1.3). The highest frequencies and RSCU values were observed for the codons ending at U and A. Apart from methionine and tryptophan which had RSCU = 1, most amino acids with numerous codons were strongly biased for one or two A/U ending codons. Stop codons in *S. sesuvioides* are UAA, UGA, and UAG (Table 3).

**Table 3:** Frequency and RSCU values of 64 codons in *S. sesuvioides* cp genome*.* Stop codons are donated by ^"*"^

Amino Acid	Codon	Freq of A.Acid	RSCU	Amino Acid	Codon	Freq of A.Acid	RSCU
^*^	UAA	1350	1.28	M	AUG	810	1
	UAG	804	0.76	N	AAU	1941	1.4
	UGA	1019	0.96		AAC	823	0.6
A	GCU	409	1.19	P	CCU	658	1.13
	GCC	345	1.01		CCC	566	0.98
	GCA	381	1.11		CCA	712	1.23
	GCG	238	0.69		CCG	384	0.66
C	UGU	660	1.21	Q	CAA	1129	1.42
	UGC	429	0.79		CAG	465	0.58
D	GAU	1006	1.44	R	CGU	382	0.7
	GAC	393	0.56		CGC	241	0.44
E	GAA	1378	1.39		CGA	644	1.18
	GAG	601	0.61		CGG	415	0.76
F	UUU	2377	1.21		AGA	989	1.81
	UUC	1544	0.79		AGG	615	1.12
G	GGU	517	0.96	S	UCU	1116	1.43
	GGC	350	0.65		UCC	898	1.15
	GGA	762	1.41		UCA	861	1.11
	GGG	535	0.99		UCG	601	0.77
H	CAU	980	1.43		AGU	683	0.88
	CAC	389	0.57		AGC	512	0.66
I	AUU	1819	1.26	T	ACU	662	1.18
	AUC	1125	0.78		ACC	589	1.05
	AUA	1374	0.95		ACA	631	1.13
K	AAA	2188	1.36		ACG	354	0.63
	AAG	1034	0.64	V	GUU	777	1.39
L	UUA	1057	1.26		GUC	451	0.81
	UUG	1107	1.32		GUA	638	1.14
	CUU	1100	1.31		GUG	374	0.67
	CUC	590	0.7	W	UGG	697	1
	CUA	708	0.84	Y	UAU	1566	1.37
	CUG	475	0.57		UAC	721	0.63

### Determination of SSRs

**Fig. 4:**
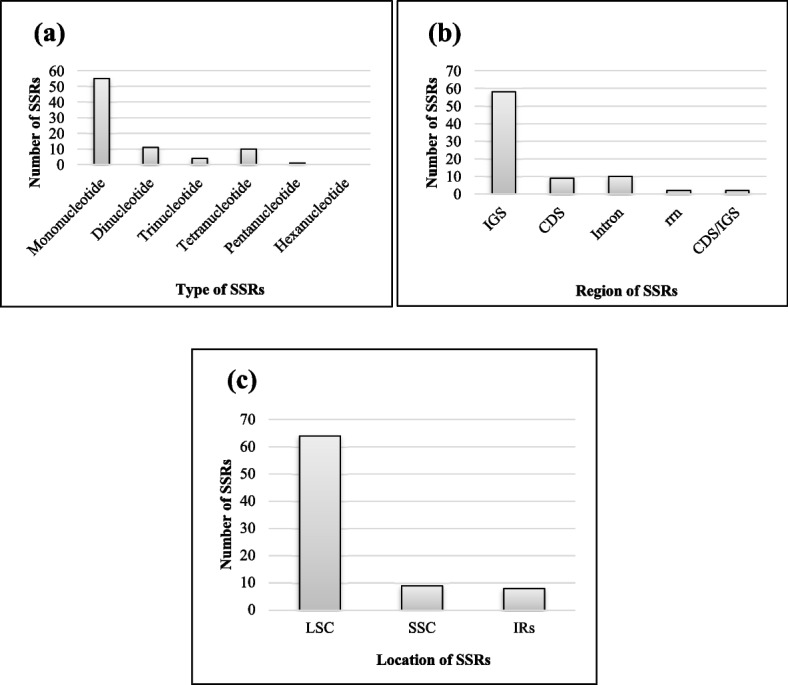
SSRs detail of cp genome of *S. sesuvioides.* **a** Types of SSRs. **b** Represent SSRs in functional cp genome regions. **c** Location of the SSRs

We discovered 81 SSRs of 19 distinct kinds in *S. sesuvioides* using the Perl script MISA (Table S1). The number of SSRs present in compound form was 7. The *S. sesuvioides* included 55 (68%) mononucleotides, 11 (14%) di-nucleotide, 4 (5%) tri-nucleotide, 10 (12%) tetra-nucleotide, and 1 (1%) pentanucleotide (Fig. 4a & Table S3). No hexanucleotides were observed in the cp genome of *S. sesuvioides*. All mononucleotide SSRs included A/T motifs, but no C/G motifs (Table S2). Di-nucleotides of four different forms have been identified i.e. AT, AT, AC, GT. In the cp genome of *S. sesuvioides*, four tri-nucleotide variants (AAT, ATT, AAC, GTT) were identified, as well as eleven tetra-nucleotide forms (AAAT, ATTT, AAAG, CTTT, AATT, AATT, AGAT, ATCT, ACCT, AGGT), and two pentanucleotide types (AAATT, AATTT). The LSC has the most SSRs (64), followed by the SSC (9), and then the inverted repeats (IRs) (8 SSRs) (Fig. 4c). The proportion of SSRs in designated areas in this scenario was as follows: 72% SSRs (58) in intergenic areas > 12% SSRs (10) in intronic regions > 11% SSRs (9) in the rrn region (Fig. 4b). We found two CDS/IGS mutual SSRs that were mononucleotides at *ycf4-cemA* and *petD-rpoA*.

### Oligonucleotide repeats analysis

**Fig. 5:**
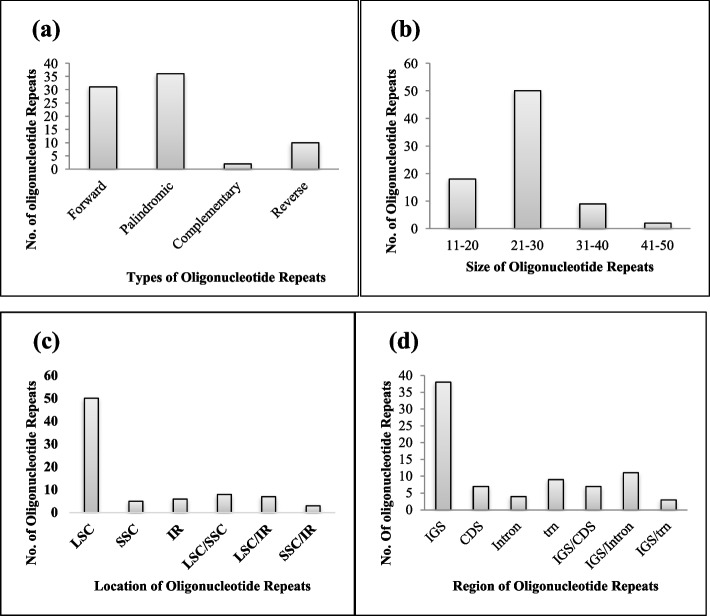
The detail of ORs of *S. sesuvioides* cp genome. **a** The amount of oligonucleotide repeats found in *S. sesuvioides* is classified into four types: reverse, complementary, palindromic, and forward repetitions. **b** Described the *S. sesuvioides* oligonucleotide repeats size range. **c** Estimation of the no. of oligonucleotide repeats in regions of the cp genome. **d** Demonstrated the functional locations of oligonucleotide repeats

We identified 79 unique oligonucleotide repeat sequences in the *S. sesuvioides* cp genome utilizing the REPuter program. We detected four types of oligonucleotide repetitions, as shown in Fig. 5(a): 31 Forward, 10 Reverse, 36 Palindromic, and two Complementary repeats. The length of the repeats varied from 19 to 50 bp (Fig. 5b). The LSC region had the most oligonucleotide repeats at 63% (50) and then IRs at 8% (6), whereas the SSC region had the lowest at 6% (5). LSC and SSC shared eight oligonucleotide repeat sequences, SSC and IR four, and LSC and IR seven (Fig. 5c). The intergenic spacer region contains the greatest oligonucleotide repeats (48%), following the trn region (11%), the CDS (9%), and the intronic region (5%). Mutual repetitions were also found in the domains CDS/IGS (9%), Intron/IGS (14%), and trn/IGS (4%), as shown in Fig. 5(d). Table S4 shows the position, arrangement, and area of the repetitions.

### Phylogenetic study of the Caryophyllales

**Fig. 6:**
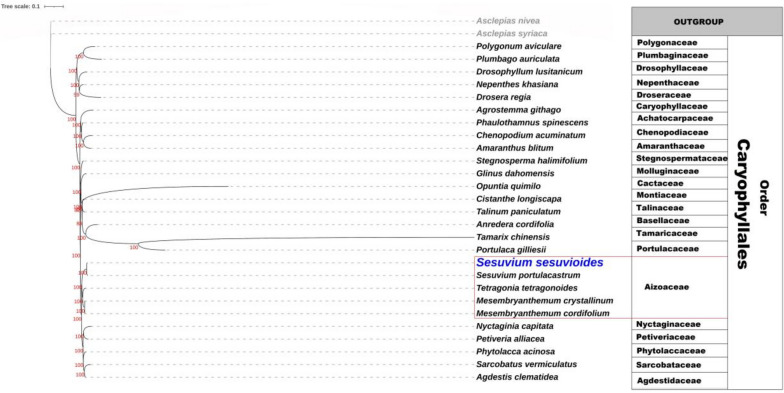
The order Caryophyllales’ maximum likelihood (ML) tree. The out-group is represented by *Asclepias nivea* and *Asclepias syriaca* (Family Apocynaceae). The genus *Sesuvium* and the genus *Tetragonia* have a close connection

There were 161,453 bp of consensus sequence nucleotide locations in the alignment of 29 species (Table S5) with a pairwise similarity of 55.7%. There were 86,947 consistent sites, 25, 280 instructive parsimony sites, and 31,809 unique site patterns discovered. The phylogenetic tree resulted in 26 branches with bootstrap values ranging from 59 to 100 (See Fig. 6). The bootstrap value for 22 among these branches was 100. The phylogenetic analysis showed the *Sesuvium* and the other two genera of the Aizoaceae family, *Tetragonia*, and *Mesembryanthemum*, were closely linked and monophyletic. A reasonable bootstrap support value for the five species of Aizoaceae was 100. The phylogenetic tree also showed that members of the Aizoaceae family were closely related to the genus *Portulaca* of the Portulacaceae family and the genus *Nyctaginia* of the Nyctaginaceae family. The Aizoaceae genera chosen were all closely connected to the *Sesuvium* genus (Fig. 6). This phylogenetic tree also highlighted the monophyly of all the represented sub-families of Aizoaceae i.e. Tetragonioideae (*Tetragonia*), Mesembryanthemoideae (*Mesembryanthemum*), Sesuvioideae (*Sesuvium*). This phylogenetic tree further demonstrated the tight connections of all Caryophyllales families, confirming the order’s monophyletic nature.

### Comparative analysis of *S. Sesuvioides* with other species

**Fig. 7:**
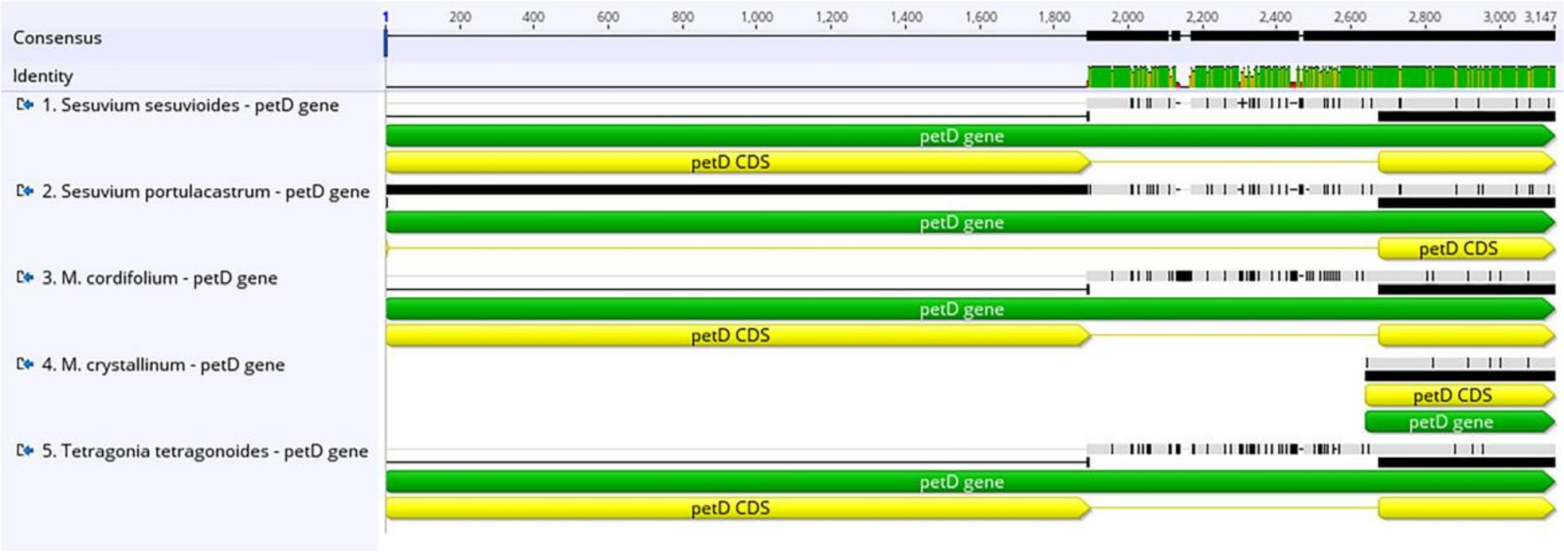
Multiple alignment *petD* gene of five species of Aizoaceae family

**Fig. 8:**
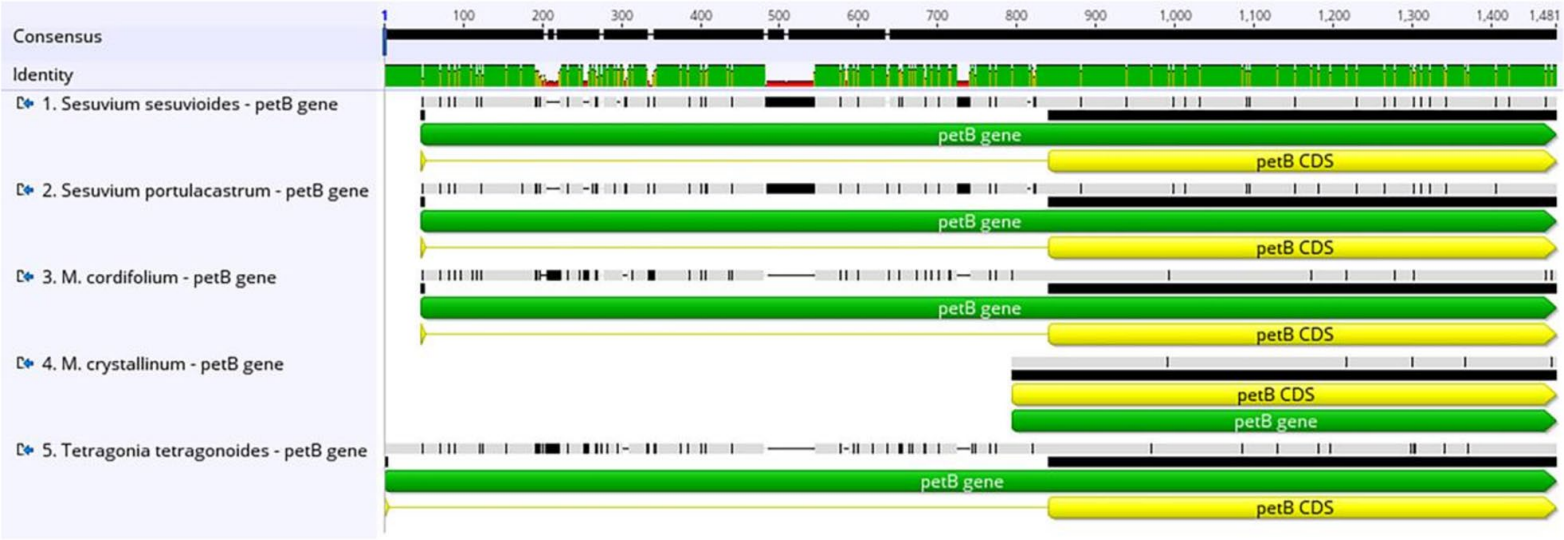
Multiple alignment *petB* gene of five species of Aizoaceae family

**Fig. 9:**
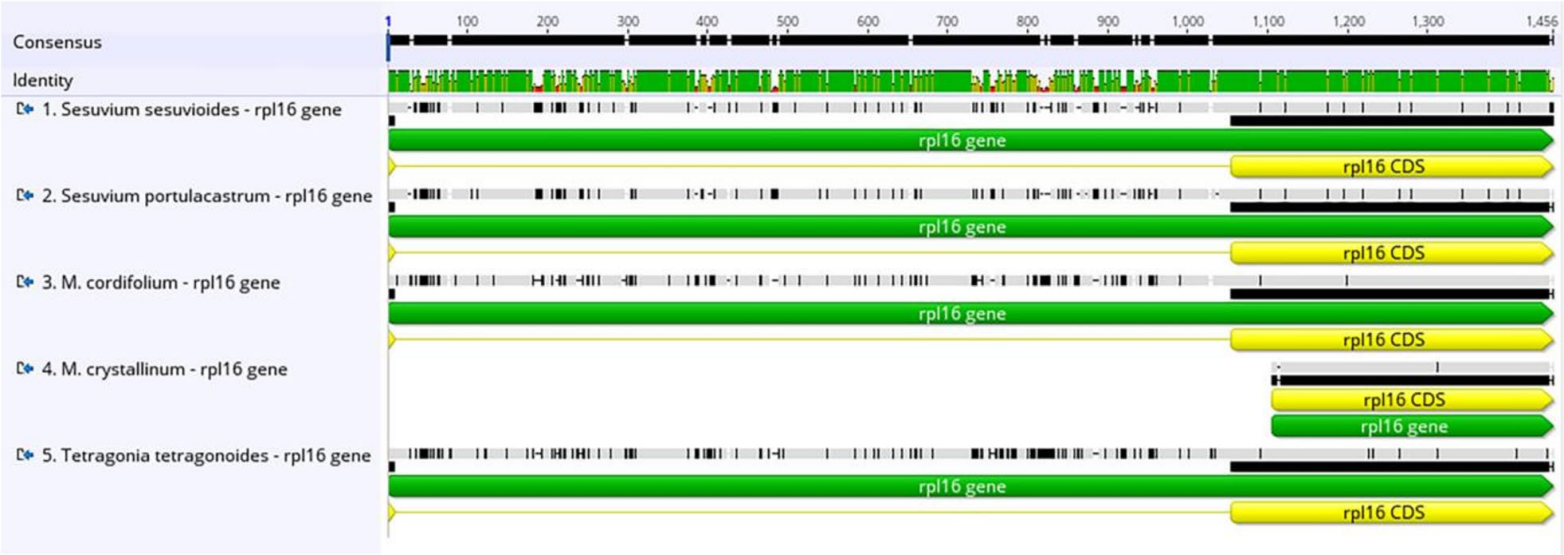
Multiple alignment *rpl16* gene of five species of Aizoaceae family

We picked four Aizoaceae species to perform a comparison with *S. sesuvioides*, including *Sesuvium portulacastrum*, *Mesembryanthemum crystallinum*, *Mesembryanthemum cordifolium*, and *Tetragonia tetragonoides*, shown in Table 4. Overall, the length of the chloroplast sequences spans from 155,934 bp (*S. portulacastrum*) to 149,506 bp (*T. tetragonoides*), and every segment of the quadripartite cycle was comparable among these cp genomes. *M. crystallinum* has the greatest number of genes (134), while *T. tetragonoides* has the fewest (127). These cp genomes’ total GC content varied between 36.6 to 37.3%. These cp genomes have similar gene compositions, with only a small number of genes being added or deleted. The *rps15* and *rpl2* introns were entirely missing in all five Aizoaceae species. The *rps19* gene has one functional copy in all genomes, but *S. sesuvioides* has an additional pseudo copy. The *infA* gene was missing in *T. tetragonoides* and was extremely small in *S. sesuvioides*. The *ycf3* gene is found only in *M. crystallinum*. The introns of the *petB*, *petD*, and *rpl16* genes are missing in *M. crystallinum* but found in the other four Aizoaceae species (Figs. 7, 8, 9). MAFFT alignment of five species was employed in Geneious Prime 2021.1.1, 162,218 bp consensus sequence had 134,189 (82.7%) identical sites and 90.5% pairwise identity.

**Table 4:** The findings of a cp genome comparison among five Aizoaceae species are shown

Genome Features	***Sesuvium sesuvioides***	***Sesuvium portulacastrum***	***Mesembryanthemum crystallinum***	***Mesembryanthemum cordifolium***	***Tetragonia tetragonoides***
Genome Size (bp)	155,849	155,934	153,831	153,722	149,506
Length of LSC (bp)	85,619	85,650	85,896	85,633	82,778
Length of SSC (bp)	18,736	18,756	18,104	18,212	17,188
Length of IR (bp)	25,747	25,764	24,916	24,915	24,770
GC content %	36.8	36.6	37.0	37.1	37.3
Total No. of genes	132	130	134	129	127
Protein Coding Genes	87	85	90	85	83
No. of tRNA genes	37	37	36	36	36
No. of rRNA genes	8	8	8	8	8
Accession Number	MW539047	MK330004	KM016695	MK397873	MF975369

### IR contraction and expansion

**Fig. 10:**
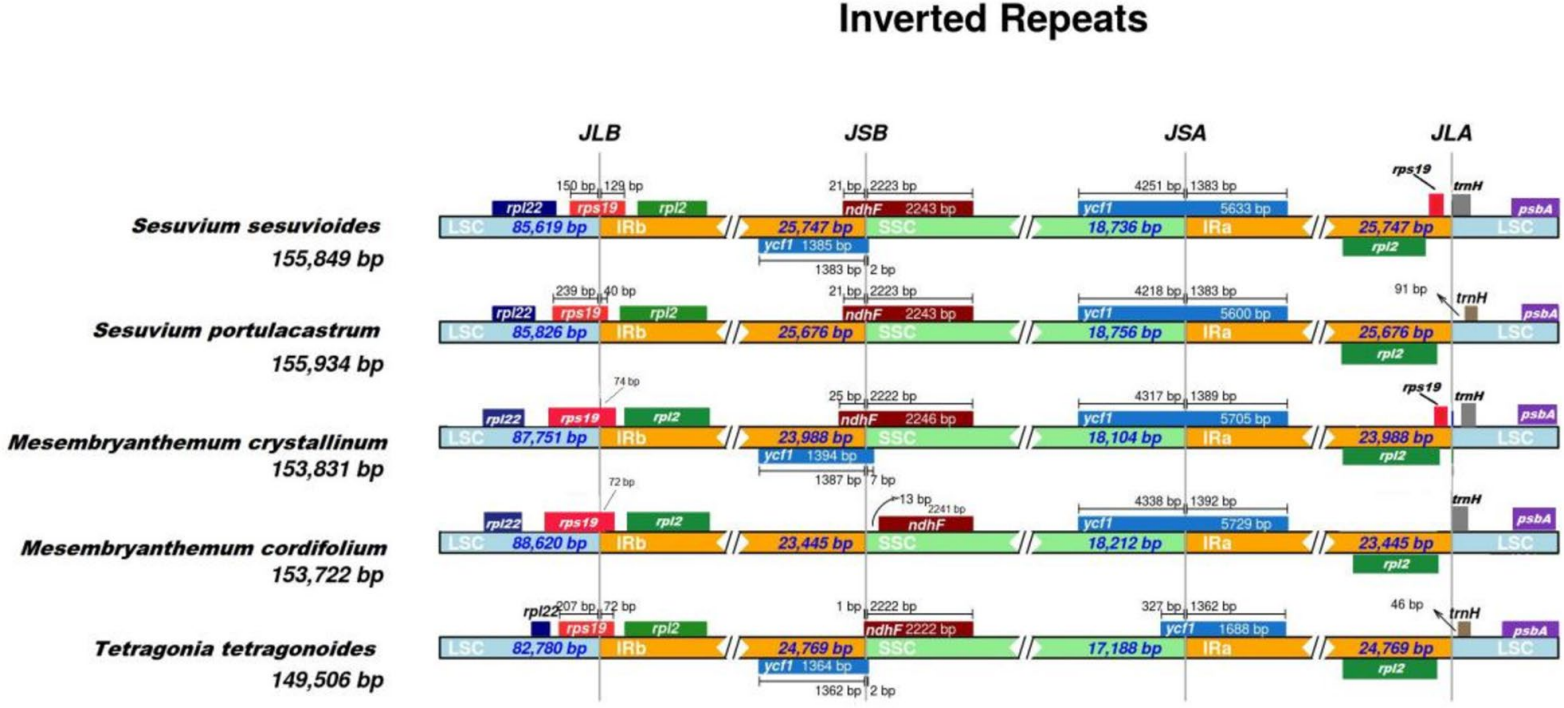
The IRSCOPE analysis of the IRs of the selected five cp genomes

Chloroplast genome evolution is influenced by variations in IR region length throughout time. In *S. sesuvioides* and four other cp genomes (*S. portulacastrum, M. crystallinum, M. cordifolium,* and *T. tetragonoides*), the contraction and expansion of IRs regions at the junctions of four main regions were examined (LSC, IRB, SSC, IRB). At the SSC/IRa border, all species contain a functional copy of the *ycf1* gene, as well as a false copy of *ycf1Ψ* at the IRb/SSC interface. In two taxa, *S. portulacastrum* and *M. cordifolium*, the *ycf1Ψ* gene was not annotated. The size of the functional *ycf1* copy spanned from 1689 to 5730 bp. The *ycf1Ψ* copy had a length that varied from 1365 to 1395 bp. Near the IRb/SSC junction, the *ndhF* gene, with a length ranging from 2139 to 2244 kb, was present in four species. The SSC region of *M. cordifolium* contains the entire *ndhF* gene. The *rps19* was located at the LSC/IRb junction in all species. The *rpl2* gene was located in the IRs. The *rpl22* was present in the LSC region of all cp genomes. At the IRa/LSC junction, the *trnH* and *psbA* genes were completely visible. These IRScope findings indicated that all Aizoaceae cp genomes are quite similar, with just minor differences in gene sizes and positions. The identification of similar genes at each chloroplast genome junction also indicated genome size correlations in these species. A thorough analysis of the contractions and expansions of IRs is shown in Fig. 10.

### The rate of Ka, and Ks substitutions

**Fig. 11:**
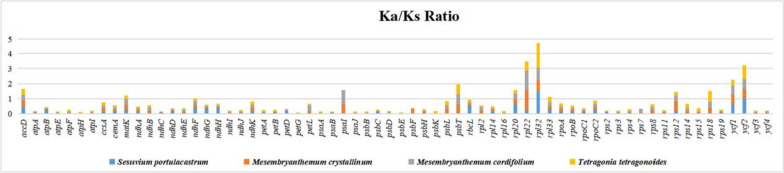
The Ka/Ks value of five Aizoaceae cp genomes. Ka/Ks values for 66 genes are mentioned; genes with 0 Ka/Ks are not presented

The Ka/Ks ratios for the *S. sesuvioides* and four Aizoaceaecp genomes were analyzed including *S. portulacastrum, M. crystallinum, M. cordifolium*, and *T. tetragonoides* (Fig. 11). *S. sesuvioides* was utilized as the reference member in each pairwise alignment. With the help of MAFFT alignment 308 pair-wise alignments on 77 related protein-coding genes were performed to examine the Ka/Ks for the selected cp genomes. Genes with irrelevant (N/A) Ka/Ks values were changed with 0 (See also Table S6). The Ka/Ks ratio varied from 0.01-1.70. The average Ka/Ks ratio was 0.212, indicating the genes underwent considerable purifying selection processes in cp genomes of the Aizoaceae family. 11 genes out of 77 (*clpP*, *petN*, *psaC*, *psbA*, *psbI*, *psbJ*, *psbM*, *psbN*, *psbZ*, *rpl23*, and *rpl36*) had Ka/Ks ratios equal to zero, confirming that these genes were preserved in all five Aizoaceae cp genomes. There were 63 genes with more synonymous substitutions (Ks) than non-synonymous substitutions (Ka). In all comparisons, all genes had lower than one Ka/Ks ratio; however, only three genes, *rpl22*, *rpl32*, and *ycf2*, showed unusual behaviour. The *ycf2* has a Ka/Ks ratio of one in *S. portulacastrum* and less than one in the other three comparisons. Similarly, the *rpl22* gene has more than one Ka/Ks value in *M. crystallinum* (1.35), and *M. cordifolium* (1.30). The *rpl32* gene has more than one Ka/Ks ratio observed in *S. portulacastrum* (1.47) and *T. tetragonoides* (1.70) while it was below one in the other two assessments (Table S6).

### Analyzing SNPs and InDel mutations in *S. Sesuvioides*

Pairwise alignments of the pertinent areas were used to compare *S. sesuvioides* to four additional Aizoaceae species to find SNPs and InDels mutations in the IRs, SSC and LSC. When *S. sesuvioides* compared to *T. tetragonoides*, had the most SNPs (7146), while *S. portulacastrum* had the fewest (1441 SNPs). A transition-to-transversion ratio greater than one resulted from the observation that the average transition rate is higher than the rate of a transversion. The Ts/Tv values in just two comparisons were less than one (Table 5). The LSC part had the greatest substitution rate, followed by the SSC and IR sections (Table S7). The LSC area had the most inDels, followed by the SSC region, and the least in the IRs (Table 6). The pairwise alignment of *S. sesuvioides* and *T. tetragonoides* produced the most inDels (11,978). The *M. cordifolium* has the second most inDels (10,148), whereas *S. portulacastrum* has the fewest (2184).

**Table 5:** Transition and Transversion substitutions, their ratio in LSC, IRs, and SSC

Region	***Pairwise alignment with Sesuvium sesuvioides***	Transition substituations	Transversion substituaions	Ts/Tv
**Large Single Copy**	*Sesuvium portulacastrum*	548	505	1.0851
	*Mesembryanthemum crystallinum*	2841	2225	1.2769
	*Mesembryanthemum cordifolium*	2840	2235	1.2707
	*Tetragonia tetragonoides*	2835	2425	1.1691
**Inverted Repeat**	*Sesuvium portulacastrum*	21	26	0.8077
	*Mesembryanthemum crystallinum*	165	124	1.3306
	*Mesembryanthemum cordifolium*	168	110	1.5273
	*Tetragonia tetragonoides*	182	192	0.9479
**Small Single Copy**	*Sesuvium portulacastrum*	178	163	1.0920
	*Mesembryanthemum crystallinum*	858	743	1.1548
	*Mesembryanthemum cordifolium*	875	763	1.1468
	*Tetragonia tetragonoides*	817	695	1.1755

**Table 6:** The detailed InDels analysis for five Aizoaceae cp genomes

Region	Species (***S. sesuvioides*** as reference)	Alignment Length	No. of InDels	InDel Average Length	InDel Diversity k(i)	InDel Diversity per site Pi(i)
Large Single Copy	*S. portulacastrum*	86,580	1891	8.294	228.000	0.00263
	*M. crystallinum*	89,438	7361	11.610	634.000	0.00709
	*M. cordifolium*	89,415	7578	12.067	628.000	0.00702
	*T. tetragonoides*	88,504	8611	13.712	628.000	0.00710
Inverted Repeat	*S. portulacastrum*	25,783	55	5.000	11.000	0.00043
	*M. crystallinum*	25,896	1129	23.041	49.000	0.00189
	*M. cordifolium*	25,908	1154	24.042	48.000	0.00185
	*T. tetragonoides*	25,875	1233	23.264	53.000	0.00205
Small Single Copy	*S. portulacastrum*	18,865	238	6.103	39.000	0.00207
	*M. crystallinum*	19,101	1362	12.270	111.000	0.00581
	*M. cordifolium*	19,182	1416	12.643	112.000	0.00584
	*T. tetragonoides*	19,029	2134	22.000	97.000	0.00510

No. Of inDels, Average InDel Length, InDel Diversity K(i), InDel Diversity per site Pi(i), and alignment length by performing pairwise alignment with four different Aizoaceae species (*S. portulacastrum, M. crystallinum, M. cordifolium,* and *T. tetragonoides)*

### The estimation of nucleotide diversity and highly polymorphic loci

**Fig. 12:**
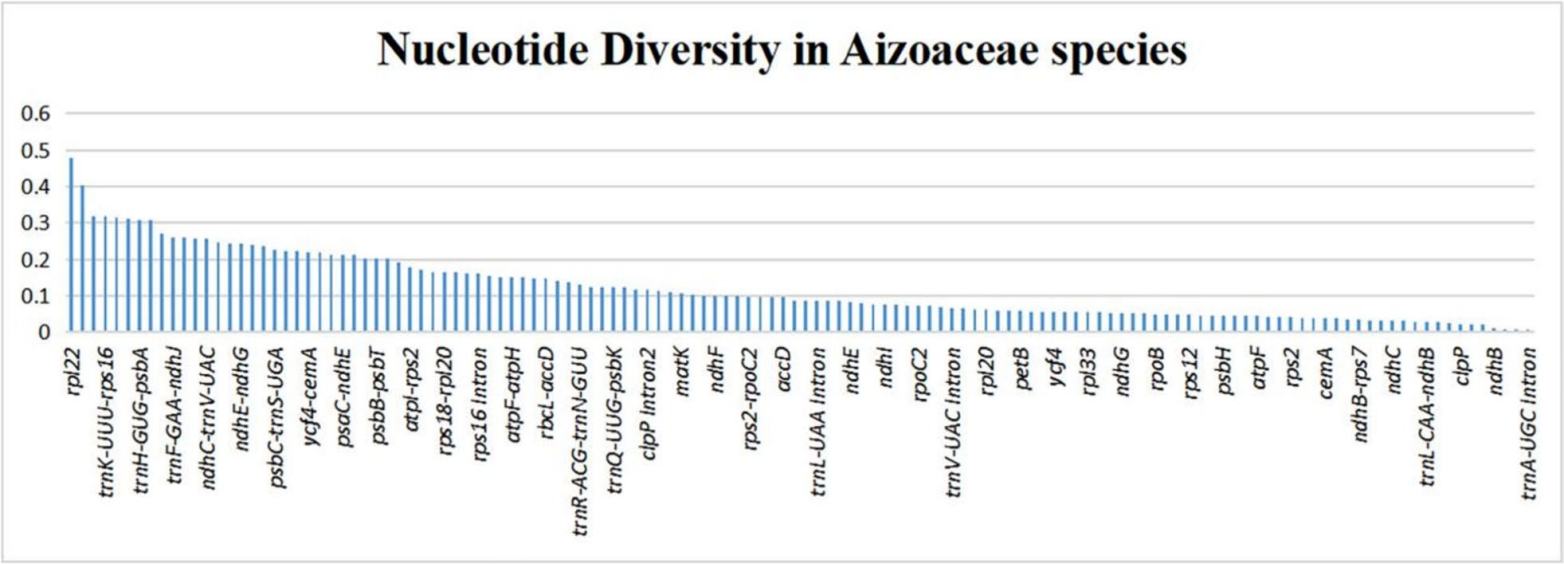
Nucleotide diversity in various areas of Aizoaceae cp genomes

Nucleotide diversity (π) was calculated in 130 common regions of *S. sesuvioides, S. portulacastrum, M. crystallinum, M. cordifolium, and T. tetragonoides* (Fig. 12). It ranged from 0.0024 to 0.4789. The *rpl22* gene has the greatest rate (0.4789) of nucleotide diversity (Table S8). Intergenic spacer areas had the maximum average nucleotide diversity (0.1913), then intronic regions (0.0865) and the lowest values for CDS regions (0.0598). Ten highly polymorphic areas were found (Table 7), nine of which were IGS polymorphic sites, and one was a protein-coding site.

**Table 7:** Ten Highly Polymorphic loci of Aizoaceae species

S.No	***Region***	Location	Nucleotide Diversity	T. No′s of Mutations	Region Length	Alignment Length
1	*rpl22*	CDS	0.478873	34	71	606
2	*trnD-GUC-trnY-GUA*	IGS	0.404145	78	193	461
3	*rpl32-trnL-UAG*	IGS	0.318426	178	559	1288
4	*trnK-UUU-rps16*	IGS	0.316294	99	313	895
5	*trnE-UUC-trnT-GGU*	IGS	0.315904	145	459	761
6	*trnM-CAU-atpE*	IGS	0.311927	68	218	295
7	*trnH-GUG-psbA*	IGS	0.308824	63	204	384
8	*psaJ-rpl33*	IGS	0.308756	134	434	524
9	*rps4-trnT-UGU*	IGS	0.268939	71	264	485
10	*trnF-GAA-ndhJ*	IGS	0.261484	74	283	723

## Discussion

The organizational structure and evolution of chloroplast genomes are useful for offering a deeper understanding of the plant genome and phylogenetic studies. This study reported on the complete cp genome of *S. sesuvioides* and compared it to the genomes of four other Aizoaceae species: *S. portulacastrum*, *M. crystallinum*, *M. cordifolium*, and *T. tetragonoides*. The plastid genome of *S. sesuvioides* was generated using advanced sequencing technology and has a typical quadripartite composition, with notable similarities to the cp genomes of *S. portulacastrum* and other Aizoaceae species [1, 50].

Leucine was shown to be the most common amino acid and cysteine is an exceptionally rare amino acid. In plants, leucine is essential for ATP production, protein synthesis, chlorophyll fluorescence modulation, tissue regeneration, net photosynthesis rate and photochemical efficiency [51, 52]. The fact that cysteine is scarce in the cp genome does not imply that it is unimportant; in fact, cysteine appears to be crucial for the redox control of the chloroplast under particular illumination circumstances [53]. The codon use bias in the cp genomes has been identified as a critical evolutionary feature for mRNA translation, new gene recognition, and molecular research [54]. In addition, certain genes’ codon usage bias in plastoms is probably a reaction to external factors [55]. All codons do not contribute equally to amino acid coding; for example, AGA coding Arginine had the largest usage bias, whereas CGC coding Arginine had the lowest. Unequal codon distribution among particular amino acids in the genome demonstrates that nucleotide mutation is not at random and that there are mutations preferred and selection pressure, leading to synonymous codon use bias [56]. Previous studies on the nucleic acid composition of many angiosperm plants [56–60] found that codons ending in amino acids U(T) and A were the most common and had the greatest relative synonymous codon usage (RSCU); our findings confirmed this. The results presented here are compatible with prior cp genomic data, suggesting that the usage bias of certain codons was caused by adaptive evolution or the configuration bias of the high A/T intensity [21, 61–63].

SSRs and oligonucleotide repeats may be useful in phylogenetics and functional genomics [21, 57, 64]. SSRs were engaged in a variety of cp genome alterations, deletions, insertions, and substantial variants [65, 66]. The most prevalent SSR type was mononucleotides, which had two repeat patterns, A and T. Several higher plants showed comparable outcomes, revealing the prevalence of adenine and thymine repeats in cp genomes [21, 57, 61, 64–66]. Hexanucleotide SSRs, which are lacking in some other cp genomes [57, 61, 65, 67] were not found in the *S. sesuvioides* genome. Sequence repeats were more abundant in single-copy sections than in inverted repeats, supporting the notion that IRs are preserved. The IGS has the most repetitions of any section of the cp genome [21, 68–70]. As a result, we concluded that IGS areas are more vulnerable to alterations and recombination of genes than protein-coding regions [71]. The abundance of palindromic sequence repeats in the cp genome suggests the occurrence of several types of similar or equivalent sequences that are either continuous or separated by a spacer region [72, 73]. Several angiosperms have a corresponding figure of SSRs and oligonucleotide repeat transmission [21, 71, 74]. These findings show that sequence repeats change chloroplast genomes and are critical for recognizing species-specific genomic diversity [75].

The cp genome sequences are an important source of data for phylogenetic studies [21, 55, 57, 76]. The emergence of scale-up sequencing methods has increased access to cp genomes with massive amounts of genetic influence [55, 63, 77]. Previously, researchers used plastomes and nuclear genome data to conduct molecular phylogenetic studies for the order Caryophyllales [2, 78–81]. According to the results, the genera *Sesuvium* and *Tetragonia* are closely related. All of the other Aizoaceae family genera are thought to be quite comparable to *Sesuvium*, confirming the family’s monophyletic nature of the family [1, 9, 82]. This phylogenetic tree also revealed that all the sub-families of the Aizoaceae family are monophyletic. Several molecular investigations, however, have established the evolutionary position and monophyly of the Aizoaceae subfamilies, namely Tetragonioideae, Aizooideae, and Sesuvioideae [29, 30, 82]. The evolutionary tree also revealed close links between all Caryophyllales families, confirming the previously stated fact that Caryophyllales is a monophyletic group based on genomic evidence [2, 78, 82]. Additionally, more comprehensive plastome data are required for a deeper comprehension of the evolutionary relationships and phylogeny of Aizoaceae.

This study found that *S. sesuvioides* and the other four Aizoaceae cp genomes had equal gene content, gene organization, GC content, and a uniform trend of intron/intron existence in the genes. Several angiosperm lineages have been demonstrated to have comparable gene organization and content for the consistent structure of the cp genome [21, 48, 71, 83]. In the Aizoaceae species, the *infA* gene was determined to be functional, non-functional, or missing from the chloroplast genome. The chloroplast genomes of many other angiosperm species have undergone gene loss and pseudogenization during evolution [81, 84–86]. The *rps15* gene and the *rpl2* intron were absent in all Aizoaceae species. However, intron loss has been seen in several angiosperm species [21, 87, 88]. The *rpoC2, atpF*, *rpl2*, *rps12*, *rps16*, and *clpP* are examples of previously known protein-coding genes with intron deletion [82, 87–90]. Introns play an important function in gene expression regulation and can increase exogenous gene expression in plant areas to attain specific agronomic characteristics [72]. The absence of particular introns may result in alterations in gene function [72]. The genes *rps12*, *ycf3*, and *clpP* exhibited multiple introns in all Aizoaceae species. More than one intron for *ycf3*, *rps12*, and *clp* was discovered in earlier chloroplast genome investigations [58–60, 91]. Additional introns have been suggested to be helpful for investigations of photosynthetic evolution [58]. The GC content is an essential measure for establishing familial connections [92, 93]. The AT content in all Aizoaceae species cp genomes was higher than the GC percentage. Our data revealed that the proportion of GC in cpDNA was not spread evenly among chloroplast genomic regions. The IR area had a higher GC content than the other areas, owing to the high GC concentration identified in the four rRNAs in the inverted repeats [94].

The IRs junctions of *S. sesuvioides* were compared to those of four other Aizoaceae species. The presence of comparable genes at similar positions in all Aizoaceae species corroborated their tight association and this study’s phylogenetic findings. Our findings are also supported by the fact that the IRs are more conserved and the majority of modifications occurred in the SSC and LSC parts [21, 68–70]. Almost all of the genes in *S. sesuvioides* and chosen Aizoaceae species were subjected to purifying selection pressure, indicating that the protein-coding genes in this family are conserved in nature. This study’s findings complement previous research findings in a variety of different angiosperms that the rate of occurrence of synonymous substitutions in protein-coding genes in cp genomes is higher than that of nonsynonymous changes [55, 75, 95, 96]. In general, synonymous substitutions are allowed in cp genomes, while nonsynonymous substitutions are eliminated by purifying selection forces, resulting in protein-coding genes being more conserved than non-coding regions [97, 98].

Throughout the majority of species comparisons with *S. sesuvioides*, the number of inDels and SNPs was greatest in the LSC and lowest in the IRs. These findings support previous findings that inDels and substitutions are much more prevalent in single-copy regions than in inverted repeats [21, 99]. We calculated a Ts/Tv ratio greater than one, which is consistent with previous research findings that the highest ratio of transitions exists at the mutational level and requires much-reduced distortions of the DNA double-helix structure than transversions, implying that transitions happen more commonly in the replication of DNA [21, 100]. The transitions tend to be less disruptive than transversions because they do not produce substantial modifications in amino acid fundamental properties [100].

To determine nucleotide diversity, we investigated IGS regions, CDS regions, and intronic regions in *S. sesuvioides* and four other Aizoaceae species. The average nucleotide diversity was smallest in coding regions and highest in intergenic spacer regions. The fact that protein-coding genes have limited nucleotide variation adds validity to the hypothesis that Aizoaceae cp genomes are relatively conservative. Nucleotide diversity varies from 0.0024 to 0.4789; lower values indicate plastome architectural retention in Aizoaceae species, and a decreased rate of nucleotide diversity has also been seen in multiple other cp genomes [21, 101–103]. Ten highly polymorphic sites with the greatest nucleotide diversity were identified as suitable molecular markers. These highly polymorphic loci might be used to create valid and reliable DNA barcodes for the *Sesuvium* and Aizoaceae family. More research into the *Sesuvium* genus and Aizoaceae family is required to corroborate these markers.

## Conclusion

The cp genome of *S. sesuvioides* is sequenced and analysed for the first time. The outcomes of this investigation will give detailed taxonomic information regarding the structure, genetic content, and evolutionary history of the Aizoaceae cp genomes. The comparison of *S. sesuvioides* to other Aizoaceae species adds to our understanding of genetic variation, substitutions, and the evolution of the family. The phylogenetic analyses of this work show significant support for the Caryophyllales order’s monophyly and the sister group connection amongst Aizoaceae subfamilies. The phylogenetic analysis will help to resolve and enhance the scant data on these family members. This study’s findings will also assist in identifying new species and creating novel molecular markers for Aizoaceae chloroplasts in plant genetic manipulation. More species must be sequenced to properly understand the Aizoaceae family’s evolutionary history.

### Supplementary Information

**Additional file 1:** Tables S1-S8.

## Data Availability

The datasets generated and/or analyzed during the current study are available in the NCBI repository, https://www.ncbi.nlm.nih.gov/nuccore/MW539047.1 ACCESSION MW539047. Raw data submitted Sequence Read Archive (SRA) under the project number PRJNA660981. Further data is present in the manuscript. For more in-depth details, there is a supplementary file.

## References

[1] Choi KS, Kwak M, Lee B, Park SJ (2018). Complete chloroplast genome of *Tetragonia tetragonioides*: molecular phylogenetic relationships and evolution in Caryophyllales. PLoS One.

[2] Brockington SF (2009). Phylogeny of the Caryophyllales sensu lato: revisiting hypotheses on pollination biology and perianth differentiation in the core Caryophyllales. Int J Plant Sci.

[3] Walker JF (2018). From cacti to carnivores: improved phylotranscriptomic sampling and hierarchical homology inference provide further insight into the evolution of Caryophyllales. Am J Bot.

[4] Klak C, Hanáček P, Bruyns PV (2017). Disentangling the Aizooideae: new generic concepts and a new subfamily in Aizoaceae. Taxon.

[5] Hernández-Ledesma P (2015). A taxonomic backbone for the global synthesis of species diversity in the angiosperm order Caryophyllales. Willdenowia.

[6] Bohley K, Schröder T, Kesselmeier J, Ludwig M, Kadereit G (2019). C 4-like photosynthesis and the effects of leaf senescence on C 4-like physiology in *Sesuvium sesuvioides* (Aizoaceae). J Exp Bot.

[7] El-Raouf HSA (2021). Taxonomic significance of leaves in family Aizoaceae. Saudi J Biol Sci.

[8] Klak C, Hanáček P, Bruyns PV (2018). A recircumscription of *Jacobsenia* (Aizoaceae): re-instating *Drosanthemopsis*, with two new quartz-endemics from Namaqualand, South Africa and sinking Knersia. South African J Bot.

[9] Liede-Schumann S, Grimm GW, Nürk NM, Potts AJ, Meve U, Hartmann HEK (2020). Phylogenetic relationships in the southern African genus *Drosanthemum* (Ruschioideae, Aizoaceae). PeerJ.

[10] Valente LM, Britton AW, Powell MP, Papadopulos AST, Burgoyne PM, Savolainen V (2014). Correlates of hyperdiversity in southern African ice plants (Aizoaceae). Bot J Linn Soc.

[11] G Bohley K, Winter PJD, Kadereit G (2017). A Revision of *Sesuvium* (Aizoaceae , Sesuvioideae). Syst Bot.

[12] Sukhorukov AP (2017). Chorological and taxonomic notes on African plants, 2. Bot Lett.

[13] Sukhorukov AP (2018). Diagnostics, taxonomy, nomenclature and distribution of perennial *Sesuvium* (Aizoaceae) in Africa. PhytoKeys.

[14] Khan AA, Chaudhry MS, Aziz S (2004). Natural resource diversity in Cholistan Desert (Pakistan) and possible conservational measures. J Pure Appl Sci.

[15] Filfilan TASA, Mehmood SF (2000). A cytological study of flowering plants from Saudi Arabia. Willdenowia.

[16] Ahmad M, Wariss S, Alam HM, Anjum K, Mukhtar S (2014). Ethnobotanical studies of plant resources of Cholistan desert, Pakistan. Int J Sci Res.

[17] Rehman F (2015). Ethnobotanical survey; common medicinal plants used by people of Cholistan Desert. Prof Med J.

[18] Javed F, Jabeen Q, Aslam N, Mehmood A (2020). Pharmacological evaluation of analgesic, anti-inflammatory and antipyretic activities of ethanolic extract of *Indigofera argentea* Burm. f. J Ethnopharmacol.

[19] Sajid-ur-Rehman M (2021). Phytochemical profiling, in vitro and in vivo anti-inflammatory, analgesic and antipyretic potential of *Sesuvium sesuvioides* (Fenzl) Verdc. (Aizoaceae). Inflammopharmacology.

[20] Freudenthal JA, Pfaff S, Terhoeven N, Korte A, Ankenbrand MJ, Förster F (2020). A systematic comparison of chloroplast genome assembly tools. Genome Biol.

[21] Javaid N, Ramzan M, Khan IA, Alahmadi TA, Datta R (2022). The chloroplast genome of *Farsetia hamiltonii* Royle, phylogenetic analysis, and comparative study with other members of clade C of Brassicaceae. BMC Plant Biol.

[22] Wicke S, Schneeweiss GM, dePamphilis CW, Müller KF, Quandt D (2011). The evolution of the plastid chromosome in land plants: gene content, gene order, gene function. Plant Mol Biol.

[23] Shinozaki K (1986). The complete nucleotide sequence of the tobacco chloroplast genome: its gene organization and expression. EMBO J.

[24] Duchene D, Bromham L. Rates of molecular evolution and diversification in plants: chloroplast substitution rates correlate with species-richness in the Proteaceae. BMC Evol Biol. 2013;13(1) 10.1186/1471-2148-13-65. 10.1186/1471-2148-13-65 PMC3600047 23497266

[25] Smith DR (2015). Mutation rates in plastid genomes: they are lower than you might think. Genome Biol Evol.

[26] Walker JF, Zanis MJ, Emery NC (2014). Comparative analysis of complete chloroplast genome sequence and inversion variation in *Lasthenia burkei* (Madieae, Asteraceae). Am J Bot.

[27] Androsiuk P (2020). Evolutionary dynamics of the chloroplast genome sequences of six *Colobanthus* species. Sci Rep.

[28] Wambugu PW, Brozynska M, Furtado A, Waters DL, Henry RJ (2015). Relationships of wild and domesticated Rices (*Oryza* AA genome species) based upon whole chloroplast genome sequences. Sci Rep.

[29] Hassan NS, Thiede J, Liede-Schumann S (2005). Phylogenetic analysis of Sesuvioideae (Aizoaceae) inferred from nrDNA internal transcribed spacer (ITS) sequences and morphological data. Plant Syst Evol.

[30] Powell RF, Magee AR, Forest F, Cowan RS, Boatwright JS (2019). A phylogeographic study of the stoneplant *Conophytum* (Aizoaceae; Ruschioideae; Ruschieae) in the Bushmanland inselberg region (South Africa) suggests anemochory. Syst Biodivers.

[31] Xia Y (2019). A modified SDS-based DNA extraction method from raw soybean. Biosci Rep.

[32] S. Andrews, “FastQC: A Quality Control Tool for High Throughput Sequence Data.” 2010. [Online]. Available: https://www.bioinformatics.babraham.ac.uk/projects/fastqc/

[33] Dierckxsens N, Mardulyn P, Smits G (2017). NOVOPlasty: De novo assembly of organelle genomes from whole genome data. Nucleic Acids Res.

[34] Tillich M (2017). GeSeq - versatile and accurate annotation of organelle genomes. Nucleic Acids Res.

[35] Shi L (2019). CPGAVAS2, an integrated plastome sequence annotator and analyzer. Nucleic Acids Res.

[36] Katoh K, Kuma KI, Toh H, Miyata T (2005). MAFFT version 5: improvement in accuracy of multiple sequence alignment. Nucleic Acids Res.

[37] Kearse M (2012). Geneious basic: an integrated and extendable desktop software platform for the organization and analysis of sequence data. Bioinformatics.

[38] Schattner P, Brooks AN, Lowe TM (2005). The tRNAscan-SE, snoscan and snoGPS web servers for the detection of tRNAs and snoRNAs. Nucleic Acids Res.

[39] Li H, Durbin R (2010). Fast and accurate long-read alignment with burrows-wheeler transform. Bioinformatics.

[40] Milne I (2009). Tablet-next generation sequence assembly visualization. Bioinformatics.

[41] Tamura K, Stecher G, Peterson D, Filipski A, Kumar S (2013). MEGA6: molecular evolutionary genetics analysis version 6.0. Mol Biol Evol.

[42] Thiel T, Michalek W, Varshney RK, Graner A (2003). Exploiting EST databases for the development and characterization of gene-derived SSR-markers in barley (Hordeum vulgare L.). Theor Appl Genet.

[43] Kurtz S, Choudhuri JV, Ohlebusch E, Schleiermacher C, Stoye J, Giegerich R (2001). REPuter: the manifold applications of repeat analysis on a genomic scale. Nucleic Acids Res.

[44] Kalyaanamoorthy S, Minh BQ, Wong TKF, Von Haeseler A, Jermiin LS (2017). ModelFinder: fast model selection for accurate phylogenetic estimates. Nat Methods.

[45] Minh BQ (2020). IQ-TREE 2: new models and efficient methods for phylogenetic inference in the genomic era. Mol Biol Evol.

[46] Hoang DT, Chernomor O, Von Haeseler A, Minh BQ, Vinh LS (2018). UFBoot2: improving the ultrafast bootstrap approximation. Mol Biol Evol.

[47] Letunic I, Bork P. Interactive tree of life (iTOL) v4: recent updates and new developments. Nucleic Acids Res. 2019;47(256–259) 10.1093/nar/gkz239. 10.1093/nar/gkz239 PMC6602468 30931475

[48] Amiryousefi A, Hyvönen J, Poczai P (2018). The chloroplast genome sequence of bittersweet (*Solanum dulcamara*): plastid genome structure evolution in Solanaceae. PLoS One.

[49] Rozas J (2017). DnaSP 6: DNA sequence polymorphism analysis of large data sets. Mol Biol Evol.

[50] Xu H (2019). The complete chloroplast genome of newly alien medicinal and toxic species, *Zaleya pentandra* (L.) C. Jeffrey (Aizoaceae), in China. Mitochondrial DNA Part B.

[51] Pedroso JAB, Zampieri TT, Donato J (2015). Reviewing the effects of l-leucine supplementation in the regulation of food intake, energy balance, and glucose homeostasis. Nutrients.

[52] Sun M, Li S, Gong Q, Xiao Y, Peng F. Leucine contributes to copper stress tolerance in peach (*Prunus persica*) seedlings by enhancing photosynthesis and the antioxidant defense system. Antioxidants. 2022;11(12) 10.3390/antiox11122455. 10.3390/antiox11122455 PMC9774504 36552663

[53] Gotor C, Álvarez C, Bermúdez MÁ, Moreno I, García I, Romero LC (2010). Low abundance does not mean less importance in cysteine metabolism. Plant Signal Behav.

[54] Song W, et al. Comparative chloroplast genome analysis of wax gourd (*Benincasa hispida*) with three Benincaseae species, revealing evolutionary dynamic patterns and phylogenetic implications. Genes (Basel). 2022;13(3) 10.3390/genes13030461. 10.3390/genes13030461 PMC8954987 35328015

[55] B. Zhu, F. Qian, Y. Hou, W. Yang, M. Cai, and X. Wu, “Complete chloroplast genome features and phylogenetic analysis of *Eruca sativa* (Brassicaceae),” PLoS One, vol. 16, no. 3 March, pp. 1–19, 2021, doi: 10.1371/journal.pone.0248556. 10.1371/journal.pone.0248556 PMC7954331 33711072

[56] Zuo LH (2017). The first complete chloroplast genome sequences of Ulmus species by de novo sequencing: genome comparative and taxonomic position analysis. PLoS One.

[57] Zarei A, Ebrahimi A, Mathur S, Lawson S (2022). The first complete chloroplast genome sequence and phylogenetic analysis of pistachio (*Pistacia vera*). Diversity.

[58] Guo S (2018). Complete chloroplast genome sequence and phylogenetic analysis of *Paeonia ostii*. Molecules.

[59] Li DM, Zhao CY, Liu XF. Complete chloroplast genome sequences of *Kaempferia galanga* and *Kaempferia elegans*: molecular structures and comparative analysis. Molecules. 2019;24(3) 10.3390/molecules24030474. 10.3390/molecules24030474 PMC6385120 30699955

[60] Zhou T (2019). The complete chloroplast genome of *Euphrasia regelii*, Pseudogenization of *ndh* genes and the phylogenetic relationships within Orobanchaceae. Front Genet.

[61] Yan C, Du J, Gao L, Li Y, Hou X (2019). The complete chloroplast genome sequence of watercress (*Nasturtium officinale* R. Br.): genome organization, adaptive evolution and phylogenetic relationships in Cardamineae. Gene.

[62] Wang Z (2020). Comparative analysis of codon usage patterns in chloroplast genomes of six Euphorbiaceae species. PeerJ.

[63] Saina JK, Gichira AW, Li ZZ, Hu GW, Wang QF, Liao K (2018). The complete chloroplast genome sequence of *Dodonaea viscosa*: comparative and phylogenetic analyses. Genetica.

[64] Shen X (2018). Complete chloroplast genome sequence and phylogenetic analysis of *Aster tataricus*. Molecules.

[65] Bi Y, Zhang MF, Xue J, Dong R, Du YP, Zhang XH (2018). Chloroplast genomic resources for phylogeny and DNA barcoding: a case study on *Fritillaria*. Sci Rep.

[66] Keller J (2017). The evolutionary fate of the chloroplast and nuclear *rps16* genes as revealed through the sequencing and comparative analyses of four novel legume chloroplast genomes from *Lupinus*. DNA Res.

[67] Hu ZY, Hua W, Huang SM, Wang HZ (2011). Complete chloroplast genome sequence of rapeseed (*Brassica napus* L.) and its evolutionary implications. Genet Resour Crop Evol.

[68] Q. jie Li *et al.*, “Chloroplast genomes elucidate diversity, phylogeny, and taxonomy of *Pulsatilla* (Ranunculaceae),” Sci Rep, vol. 10, no. 1, pp. 1–12, 2020, doi: 10.1038/s41598-020-76699-7. 10.1038/s41598-020-76699-7 PMC7666119 33188288

[69] Cao J, et al. Development of chloroplast genomic resources in Chinese yam (*Dioscorea polystachya*). Biomed Res Int. 2018:1–11. 10.1155/2018/6293847. 10.1155/2018/6293847 PMC5872661 29725599

[70] Liu L (2018). Chloroplast genome analyses and genomic resource development for epilithic sister genera *Oresitrophe* and *Mukdenia* (Saxifragaceae), using genome skimming data. BMC Genomics.

[71] Menezes APA (2018). Chloroplast genomes of *Byrsonima* species (Malpighiaceae): comparative analysis and screening of high divergence sequences. Sci Rep.

[72] Liang C (2019). A comparative analysis of the chloroplast genomes of four *Salvia* medicinal plants. Engineering.

[73] Alzahrani D, Albokhari E, Yaradua S, Abba A (2021). Complete chloroplast genome sequences of *Dipterygium glaucum* and *Cleome chrysantha* and other Cleomaceae species, comparative analysis and phylogenetic relationships. Saudi J Biol Sci.

[74] Saina JK, Li ZZ, Gichira AW, Liao YY. The complete chloroplast genome sequence of tree of heaven (*Ailanthus altissima* (mill.)) (sapindales: Simaroubaceae), an important pantropical tree. Int J Mol Sci. 2018;19(4) 10.3390/ijms19040929. 10.3390/ijms19040929 PMC5979363 29561773

[75] Du X (2020). The complete chloroplast genome sequence of Yellow Mustard (*Sinapis alba* L.) and its phylogenetic relationship to other Brassicaceae species. Gene.

[76] Yu X, Tan W, Zhang H, Gao H, Wang W, Tian X (2019). Complete chloroplast genomes of *Ampelopsis humulifolia* and *Ampelopsis japonica*: molecular structure, comparative analysis, and phylogenetic analysis. Plants.

[77] Saarela JM (2018). A 250 plastome phylogeny of the grass family (Poaceae): topological support under different data partitions. PeerJ.

[78] Cuénoud P, Savolainen V, Chatrou LW, Powell M, Grayer RJ, Chase MW (2002). Molecular phylogenetics of Caryophyllales based on nuclear 18S rDNA and plastid *rbcL*, *atpB*, and *matK* DNA sequences. Am J Bot.

[79] Ruhlman T (2006). Complete plastid genome sequence of *Daucus carota*: implications for biotechnology and phylogeny of angiosperms. BMC Genomics.

[80] Wang X, Zhou T, Bai G, Zhao Y (2018). Complete chloroplast genome sequence of *Fagopyrum dibotrys*: genome features, comparative analysis and phylogenetic relationships. Sci Rep.

[81] Scobeyeva VA, et al. Gene loss, pseudogenization in plastomes of genus *Allium* (Amaryllidaceae), and putative selection for adaptation to environmental conditions. Front Genet. 2021;12(July) 10.3389/fgene.2021.674783. 10.3389/fgene.2021.674783 PMC8296844 34306019

[82] Downie SR, Palmer JD (1994). A chloroplast DNA phylogeny of the Caryophyllales based on structural and inverted repeat restriction site variation. Syst Bot.

[83] Li W (2018). Interspecific chloroplast genome sequence diversity and genomic resources in *Diospyros*. BMC Plant Biol.

[84] Li Y (2017). Gene losses and partial deletion of small single-copy regions of the chloroplast genomes of two hemiparasitic *Taxillus* species. Sci Rep.

[85] Frailey DC, Chaluvadi SR, Vaughn JN, Coatney CG, Bennetzen JL (2018). Gene loss and genome rearrangement in the plastids of five Hemiparasites in the family Orobanchaceae. BMC Plant Biol.

[86] Millen RS (2001). Many parallel losses of *infA* from chloroplast DNA during angiosperm evolution with multiple independent transfers to the nucleus. Plant Cell.

[87] Sloan DB, Triant DA, Forrester NJ, Bergner LM, Wu M, Taylor DR (2014). A recurring syndrome of accelerated plastid genome evolution in the angiosperm tribe Sileneae (Caryophyllaceae). Mol Phylogenet Evol.

[88] Jansen RK, Wojciechowski MF, Sanniyasi E, Lee SB, Daniell H (2008). Complete plastid genome sequence of the chickpea (*Cicer arietinum*) and the phylogenetic distribution of *rps12* and *clpP* intron losses among legumes (Leguminosae). Mol Phylogenet Evol.

[89] He P, Huang S, Xiao G, Zhang Y, Yu J (2016). Abundant RNA editing sites of chloroplast protein-coding genes in *Ginkgo biloba* and an evolutionary pattern analysis. BMC Plant Biol.

[90] Jansen RK (2007). Analysis of 81 genes from 64 plastid genomes resolves relationships in angiosperms and identifies genome-scale evolutionary patterns. Proc Natl Acad Sci U S A.

[91] Song Y, Chen Y, Lv J, Xu J, Zhu S, Li M. Comparative chloroplast genomes of *Sorghum* species: sequence divergence and phylogenetic relationships. Biomed Res Int. 2019;2019 10.1155/2019/5046958. 10.1155/2019/5046958 PMC6444266 31016191

[92] Asaf S (2017). Chloroplast genomes of *Arabidopsis halleri* ssp. *gemmifera* and *Arabidopsis lyrata* ssp. *petraea*: structures and comparative analysis. Sci Rep.

[93] Xu C (2017). Comparative analysis of six *Lagerstroemia* complete chloroplast genomes. Front Plant Sci.

[94] Zhang Z (2022). Characterization of the complete chloroplast genome of *Brassica oleracea* var. *italica* and phylogenetic relationships in Brassicaceae. PLoS One.

[95] Odago WO (2022). Analysis of the complete Plastomes of 31 species of *Hoya* group: insights into their comparative genomics and phylogenetic relationships. Front Plant Sci.

[96] Huang CH (2016). Resolution of Brassicaceae phylogeny using nuclear genes uncovers nested radiations and supports convergent morphological evolution. Mol Biol Evol.

[97] Lawrie DS, Messer PW, Hershberg R, Petrov DA (2013). Strong purifying selection at synonymous sites in *D. Melanogaster*. PLoS Genet.

[98] Firetti F, Zuntini AR, Gaiarsa JW, Oliveira RS, Lohmann LG. Complete chloroplast genome sequences contribute to plant species delimitation : A case study of the *Anemopaegma* species complex 1. 2017;104(10):1493–509. 10.3732/ajb.1700302. 10.3732/ajb.1700302 29885220

[99] Kim HT, Kim KJ (2014). Chloroplast genome differences between Asian and American *Equisetum arvense* (Equisetaceae) and the origin of the hypervariable *trnY-trnE* intergenic spacer. PLoS One.

[100] Zou Z, Zhang J (2021). Are nonsynonymous transversions generally more deleterious than nonsynonymous transitions?. Mol Biol Evol.

[101] Odintsova MS, Yurina NP (2003). Plastid genomes of higher plants and algae: structure and functions. Mol Biol.

[102] Cai J, Ma PF, Li HT, Li DZ (2015). Complete plastid genome sequencing of four *Tilia* species (Malvaceae): a comparative analysis and phylogenetic implications. PLoS One.

[103] Smith DR, Keeling PJ (2015). Mitochondrial and plastid genome architecture: reoccurring themes, but significant differences at the extremes. Proc Natl Acad Sci U S A.

