# Supplementary material for: Genomic exploration of Sesuvium sesuvioides: comparative study and phylogenetic analysis within the order Caryophyllales from Cholistan desert, Pakistan
Source: BMC Plant Biol. 2023 Dec 20;23:658. doi: 10.1186/s12870-023-04670-5 (PMC10731703; doi:10.1186/s12870-023-04670-5)
Supplement: Supplementary file 1 — Additional file 1: Tables S1-S8. [file 12870_2023_4670_MOESM1_ESM.docx]

**Genomic exploration of *Sesuvium sesuvioides*: comparative study and phylogenetic analysis within the order Caryophyllales from Cholistan desert, Pakistan**

Nida Javaid^1^, Musarrat Ramzan^1^*, Shagufta Jabeen^2^, Subhan Danish^3^

^1^ The Islamia University Bahawalpur, Bahawalpur, Punjab, Pakistan; [nidajatala1@gmail.com](mailto:nidajatala1@gmail.com); [musarrat.ramzan@iub.edu.pk](mailto:musarrat.ramzan@iub.edu.pk);

^2^Government Associate College for Women Ahmedpur East, Bahawalpur, Punjab Pakistan; [shaguftaiub5@gmail.com](mailto:shaguftaiub5@gmail.com)

^3^Department of Soil Science, Faculty of Agricultural Sciences and Technology, Bahauddin Zakariya University, Multan, Punjab, Pakistan; [sd96850@gmail.com](mailto:sd96850@gmail.com)

Corresponding author: [musarrat.ramzan@iub.edu.pk](mailto:musarrat.ramzan@iub.edu.pk); [sd96850@gmail.com](mailto:sd96850@gmail.com)

***Supplementary Material***

1. **Supplementary tables**

**Supplementary Table 1**: Simple sequence repeats types, size, and location in *S. sesuvioides.*

| **SSR type** | **SSR** | **Size** | **Start** | **End** | **Region** | **Locus** | ***Location*** |
| --- | --- | --- | --- | --- | --- | --- | --- |
| p2 | (AT)5 | 10 | 294 | 303 | LSC | IGS | *trnH-GUG-psbA* |
| p5 | (TAAAT)3 | 15 | 4440 | 4454 | LSC | IGS | *trnK-UUU-rps16* |
| p3 | (TTA)4 | 12 | 4764 | 4775 | LSC | IGS | *trnK-UUU-rps16* |
| p1 | (A)10 | 10 | 6466 | 6475 | LSC | IGS | *rps16-trnQ-UUG* |
| p1 | (A)10 | 10 | 7455 | 7464 | LSC | IGS | *trnQ-UUG-psbK* |
| p1 | (A)13 | 13 | 8077 | 8089 | LSC | IGS | *psbK-psbI* |
| p1 | (A)10 | 10 | 8326 | 8335 | LSC | IGS | *psbI-trnS-GCU* |
| c | (A)12(AT)5 | 106 | 8493 | 8598 | LSC | IGS | *trnS-GCU-trnS-CGA* |
| c | (T)11(A)13 | 25 | 9008 | 9032 | LSC | IGS | *trnS-GCU-trnS-CGA* |
| p1 | (T)11 | 11 | 9437 | 9447 | LSC | Intron | *trnS-CGA* |
| p1 | (T)10 | 10 | 10316 | 10325 | LSC | IGS | *trnR-UCU-apA* |
| p1 | (T)10 | 10 | 12668 | 12677 | LSC | Intron | *atpF* |
| p1 | (A)16 | 16 | 13935 | 13950 | LSC | IGS | *atpH-atpI* |
| p2 | (AT)6 | 12 | 15511 | 15522 | LSC | IGS | *atpI-rps2* |
| p1 | (T)10 | 10 | 16334 | 16343 | LSC | IGS | *rps2-rpoC2* |
| p1 | (T)11 | 11 | 18554 | 18564 | LSC | CDS | *rpoC2* |
| p2 | (AT)5 | 10 | 19904 | 19913 | LSC | CDS | *rpoC2* |
| p1 | (T)13 | 13 | 22694 | 22706 | LSC | Intron | *rpoC1* |
| p1 | (T)10 | 10 | 26307 | 26316 | LSC | CDS | *rpoB* |
| c | (A)10(A)10 | 47 | 30188 | 30234 | LSC | IGS | *psbM-trnD-GUC* |
| c | (TA)5(T)12 | 59 | 31578 | 31636 | LSC | IGS | *trnE-UUC-trnT-GGU* |
| p2 | (TA)6 | 12 | 31919 | 31930 | LSC | IGS | *trnE-UUC-trnT-GGU* |
| p4 | (TCTT)3 | 12 | 32872 | 32883 | LSC | IGS | *trnT-GGU-psbD* |
| p1 | (T)11 | 11 | 33257 | 33267 | LSC | IGS | *trnT-GGU-psbD* |
| p1 | (A)10 | 10 | 37084 | 37093 | LSC | IGS | *trnG-GCC-trnfM-CAU* |
| p1 | (T)16 | 16 | 42541 | 42556 | LSC | IGS | *psaA-ycf3* |
| p1 | (T)11 | 11 | 42974 | 42984 | LSC | IGS | *psaA-ycf3* |
| p1 | (A)10 | 10 | 45154 | 45163 | LSC | Intron | *ycf3* |
| p1 | (A)13 | 13 | 45620 | 45632 | LSC | IGS | *ycf3-trnS-GGA* |
| p2 | (TA)5 | 10 | 46873 | 46882 | LSC | IGS | *rps4-trnT-UGU* |
| p2 | (TA)5 | 10 | 47023 | 47032 | LSC | IGS | *rps4-trnT-UGU* |
| p4 | (TATC)3 | 12 | 47629 | 47640 | LSC | IGS | *trnT-UGU-trnL-UAA* |
| p2 | (AT)5 | 10 | 48998 | 49007 | LSC | IGS | *trnF-GAA-ndhJ* |
| p1 | (T)11 | 11 | 51585 | 51595 | LSC | IGS | *ndhC-trnV-UAC* |
| p1 | (T)10 | 10 | 55376 | 55385 | LSC | CDS | *atpB* |
| p3 | (TTG)4 | 12 | 55551 | 55562 | LSC | IGS | *atpB-rbcL* |
| p3 | (TAT)4 | 12 | 57855 | 57866 | LSC | IGS | *rbcL-accD* |
| p4 | (ATTA)4 | 16 | 60358 | 60373 | LSC | IGS | *accD-psaI* |
| p1 | (A)10 | 10 | 60658 | 60667 | LSC | IGS | *psaI-ycf4* |
| p1 | (A)10 | 10 | 62056 | 62065 | LSC | IGS | *ycf4-cemA* |
| p1 | (A)11 | 11 | 62177 | 62187 | LSC | IGS/CDS | *ycf4-cemA* |
| p1 | (T)14 | 14 | 64185 | 64198 | LSC | IGS | *petA-psbJ* |
| c | (T)14(TTA)4 | 38 | 64606 | 64643 | LSC | IGS | *petA-psbJ* |
| c | (ATAA)3(T)10 | 95 | 66525 | 66619 | LSC | IGS | *psbE-petL* |
| p1 | (T)10 | 10 | 67227 | 67236 | LSC | IGS | *petL-petG* |
| p1 | (A)18 | 18 | 68011 | 68028 | LSC | IGS | *trnP-UGG-psaJ* |
| p1 | (A)15 | 15 | 68794 | 68808 | LSC | IGS | *psaJ-rpl33* |
| p4 | (TTTA)3 | 12 | 69812 | 69823 | LSC | IGS | *rps18-rpl20* |
| p1 | (T)12 | 12 | 71906 | 71917 | LSC | Intron | *clpP* |
| p1 | (T)10 | 10 | 72625 | 72634 | LSC | Intron | *clpP* |
| p2 | (TG)5 | 10 | 72879 | 72888 | LSC | Intron | *clpP* |
| p1 | (A)12 | 12 | 73596 | 73607 | LSC | IGS | *clpP-psbB* |
| p2 | (TA)8 | 16 | 78415 | 78430 | LSC | Intron | *petD* |
| p1 | (T)10 | 10 | 79341 | 79350 | LSC | IGS/CDS | *petD-rpoA* |
| p1 | (T)10 | 10 | 79565 | 79574 | LSC | CDS | *rpoA* |
| p1 | (T)12 | 12 | 82490 | 82501 | LSC | IGS | *rpl14-rpl16* |
| p1 | (T)10 | 10 | 83609 | 83618 | LSC | Intron | *rps16* |
| p4 | (TTTC)3 | 12 | 83742 | 83753 | LSC | Intron | *rps16* |
| p1 | (T)12 | 12 | 85768 | 85779 | IRB | IGS | *rps19-rpl2* |
| p1 | (T)11 | 11 | 100727 | 100737 | IRB | IGS | *rps12-trnV-GAC* |
| p4 | (AGGT)3 | 12 | 106930 | 106941 | IRB | rrn | *rrn23* |
| p1 | (T)14 | 14 | 109104 | 109117 | IRB | IGS | *trnR-ACG-trnN-GUU* |
| p1 | (A)10 | 10 | 113766 | 113775 | SSC | IGS | *ndhF-rpl32* |
| p1 | (A)16 | 16 | 115134 | 115149 | SSC | IGS | *rpl32-trnL-UAG* |
| p1 | (A)13 | 13 | 116061 | 116073 | SSC | IGS | *rpl32-trnL-UAG* |
| p1 | (T)10 | 10 | 125545 | 125554 | SSC | IGS | *ndhH-ycf1* |
| p1 | (A)12 | 12 | 125673 | 125684 | SSC | IGS | *ndhH-ycf1* |
| p1 | (T)10 | 10 | 127044 | 127053 | SSC | CDS | *ycf1* |
| c | (AAAT)3(TAAT)3 | 123 | 127413 | 127535 | SSC | CDS | *ycf1* |
| p1 | (T)17 | 17 | 128524 | 128540 | SSC | CDS | *ycf1* |
| p1 | (A)14 | 14 | 132352 | 132365 | IRA | IGS | *trnN-GUU-trnR-ACG* |
| p4 | (CTAC)3 | 12 | 134526 | 134537 | IRA | rrn | *rrn23* |
| p1 | (A)11 | 11 | 140732 | 140742 | IRA | IGS | *trnV-GAC-rps12* |
| p1 | (A)12 | 12 | 155690 | 155701 | IRA | IGS | *rpl2-rps19* |

**Supplementary Table 2**: Types and the number of simple sequence repeats motifs in *S. sesuvioides* cp genome*.*

| **Repeats** | **3** | **4** | **5** | **6** | **7** | **8** | **9** | **10** | **11** | **12** | **13** | **14** | **15** | **16** | **17** | **18** | **19** | **20** | **Total** |
| --- | --- | --- | --- | --- | --- | --- | --- | --- | --- | --- | --- | --- | --- | --- | --- | --- | --- | --- | --- |
| A | - | - | - | - | - | - | - | 10 | 2 | 4 | 4 | 1 | 1 | 2 |  | 1 |  |  | 25 |
| T | - | - | - | - | - | - | - | 13 | 7 | 4 | 1 | 3 |  | 1 | 1 |  |  |  | 30 |
| AT | - | - | 4 | 1 |  |  |  |  |  |  |  |  |  |  |  |  |  |  | 5 |
| TA | - | - | 3 | 1 |  | 1 |  |  |  |  |  |  |  |  |  |  |  |  | 5 |
| TG | - | - | 1 |  |  |  |  |  |  |  |  |  |  |  |  |  |  |  | 1 |
| TAT | - | 1 |  |  |  |  |  |  |  |  |  |  |  |  |  |  |  |  | 1 |
| TTA | - | 2 |  |  |  |  |  |  |  |  |  |  |  |  |  |  |  |  | 2 |
| TTG | - | 1 |  |  |  |  |  |  |  |  |  |  |  |  |  |  |  |  | 1 |
| AAAT | 1 |  |  |  |  |  |  |  |  |  |  |  |  |  |  |  |  |  | 1 |
| AGGT | 1 |  |  |  |  |  |  |  |  |  |  |  |  |  |  |  |  |  | 1 |
| ATAA | 1 |  |  |  |  |  |  |  |  |  |  |  |  |  |  |  |  |  | 1 |
| ATTA |  | 1 |  |  |  |  |  |  |  |  |  |  |  |  |  |  |  |  | 1 |
| CTAC | 1 |  |  |  |  |  |  |  |  |  |  |  |  |  |  |  |  |  | 1 |
| TAAT | 1 |  |  |  |  |  |  |  |  |  |  |  |  |  |  |  |  |  | 1 |
| TATC | 1 |  |  |  |  |  |  |  |  |  |  |  |  |  |  |  |  |  | 1 |
| TCTT | 1 |  |  |  |  |  |  |  |  |  |  |  |  |  |  |  |  |  | 1 |
| TTTA | 1 |  |  |  |  |  |  |  |  |  |  |  |  |  |  |  |  |  | 1 |
| TTTC | 1 |  |  |  |  |  |  |  |  |  |  |  |  |  |  |  |  |  | 1 |
| TAAAT | 1 |  |  |  |  |  |  |  |  |  |  |  |  |  |  |  |  |  | 1 |

**Supplementary Table 3:** Frequency of complementary simple sequence repeats in *S. sesuvioides*.

| **Repeats** | **3** | **4** | **5** | **6** | **7** | **8** | **9** | **10** | **11** | **12** | **13** | **14** | **15** | **16** | **17** | **18** | **19** | **20** | **Total** |
| --- | --- | --- | --- | --- | --- | --- | --- | --- | --- | --- | --- | --- | --- | --- | --- | --- | --- | --- | --- |
| A/T | - | - | - | - | - | - | - | 23 | 9 | 8 | 5 | 4 | 1 | 3 | 1 | 1 |  |  | 55 |
| AC/GT | - | - | 1 |  |  |  |  |  |  |  |  |  |  |  |  |  |  |  | 1 |
| AT/AT | - | - | 7 | 2 |  | 1 |  |  |  |  |  |  |  |  |  |  |  |  | 10 |
| AAC/GTT | - | 1 |  |  |  |  |  |  |  |  |  |  |  |  |  |  |  |  | 1 |
| AAT/ATT | - | 3 |  |  |  |  |  |  |  |  |  |  |  |  |  |  |  |  | 3 |
| AAAG/CTTT | 2 |  |  |  |  |  |  |  |  |  |  |  |  |  |  |  |  |  | 2 |
| AAAT/ATTT | 3 |  |  |  |  |  |  |  |  |  |  |  |  |  |  |  |  |  | 3 |
| AATT/AATT | 1 | 1 |  |  |  |  |  |  |  |  |  |  |  |  |  |  |  |  | 2 |
| ACCT/AGGT | 2 |  |  |  |  |  |  |  |  |  |  |  |  |  |  |  |  |  | 2 |
| AGAT/ATCT | 1 |  |  |  |  |  |  |  |  |  |  |  |  |  |  |  |  |  | 1 |
| AAATT/AATTT | 1 |  |  |  |  |  |  |  |  |  |  |  |  |  |  |  |  |  | 1 |

**Supplementary Table 4**: Oligo-repeats analysis in *S. sesuvioides,* their location, region, type, and lengths.

| **Size** | **Position 1** | **Type** | **Position 2** | **Location** | **Region** | **IGS/CDS/Intron** |
| --- | --- | --- | --- | --- | --- | --- |
| 22 | 278 | P | 278 | *trnH-GUG-psbA* | LSC | IGS |
| 19 | 287 | C | 46866 | *trnH-GUG-psbA/rps4-trnT-UGU* | LSC | IGS |
| 23 | 287 | P | 78408 | *trnH-GUG-psbA/petD* | LSC | IGS/Intron |
| 19 | 289 | R | 289 | *trnH-GUG-psbA* | LSC | IGS |
| 36 | 4479 | F | 114442 | *trnK-UUU-rps16/ndhF-rpl32* | LSC/SSC | IGS |
| 25 | 4486 | F | 114453 | *trnK-UUU-rps16/ndhF-rpl32* | LSC/SSC | IGS |
| 24 | 4601 | R | 22678 | *trnK-UUU-rps16/rpoC1* | LSC | IGS/Intron |
| 25 | 4689 | P | 4689 | *trnK-UUU-rps16* | LSC | IGS |
| 21 | 4753 | R | 4753 | *trnK-UUU-rps16* | LSC | IGS |
| 38 | 4819 | P | 4820 | *trnK-UUU-rps16* | LSC | IGS |
| 23 | 5013 | F | 5035 | *trnK-UUU-rps16* | LSC | IGS |
| 27 | 8065 | F | 48011 | *psbK-psbI/trnL-UAA* | LSC | IGS/Intron |
| 21 | 8068 | F | 9011 | *psbK-psbI/trnS-GCU-trnS-CGA* | LSC | IGS |
| 28 | 8240 | R | 122736 | *psbI-trnS-GCU/ndhA* | LSC/SSC | IGS/Intron |
| 24 | 8244 | R | 122736 | *psbI-trnS-GCU/ndhA* | LSC/SSC | IGS/Intron |
| 29 | 8274 | P | 8515 | *psbI-trnS-GCU/trnS-GCU-trnS-CGA* | LSC | IGS |
| 30 | 8391 | P | 45845 | *psbI-trnS-GCU/trnS-GGA* | LSC | IGS/trn |
| 25 | 8393 | F | 36033 | *trnS-GCU/trnS-UGA* | LSC | trn |
| 21 | 8397 | F | 36037 | *trnS-GCU/trnS-UGA* | LSC | trn |
| 19 | 8539 | P | 14173 | *trnS-GCU-trnS-CGA/atpH-atpI* | LSC | IGS |
| 31 | 8999 | P | 9007 | *trnS-GCU-trnS-CGA* | LSC | IGS |
| 30 | 9014 | R | 68005 | *trnS-GCU-trnS-CGA/trnP-UGG-psaJ* | LSC | IGS |
| 23 | 9019 | R | 68005 | *trnS-GCU-trnS-CGA/trnP-UGG-psaJ* | LSC | IGS |
| 19 | 9019 | P | 109097 | *trnS-GCU-trnS-CGA/trnR-ACG-trnN-GUU* | LSC/IR | IGS |
| 23 | 9020 | R | 68005 | *trnS-GCU-trnS-CGA/trnP-UGG-psaJ* | LSC | IGS |
| 25 | 9983 | F | 37029 | *trnS-CGA/trnG-GCC* | LSC | trn |
| 19 | 13934 | F | 115133 | *atpH-atpI/rpl32-trnL-UAG* | LSC/SSC | IGS |
| 27 | 22558 | F | 83310 | *rpoC1/rpl15* | LSC | Intron |
| 20 | 22586 | P | 22586 | *rpoC1* | LSC | Intron |
| 19 | 22685 | R | 42973 | *rpoC1/psaA-ycf3* | LSC | IGS/Intron |
| 20 | 25373 | F | 32849 | *rpoB/trnT-GGU-psbD* | LSC | CDS/IGS |
| 25 | 26299 | P | 30216 | *rpoB/psbM-trnD-GUC* | LSC | CDS/IGS |
| 23 | 30893 | P | 30923 | *trnD-GUC-trnY-GUA* | LSC | IGS |
| 28 | 31553 | C | 68506 | *trnE-UUC-trnT-GGU/psaJ-rpl33* | LSC | IGS |
| 22 | 31568 | P | 36254 | *trnE-UUC-trnT-GGU/trnS-UGA-psbZ* | LSC | IGS |
| 19 | 31617 | P | 68796 | *trnE-UUC-trnT-GGU/psaJ-rpl33* | LSC | IGS |
| 27 | 32178 | P | 32178 | *trnT-GGU-psbD* | LSC | IGS |
| 25 | 36033 | P | 45848 | *trnS-UGA/trnS-GGA* | LSC | trn |
| 21 | 36037 | P | 45848 | *trnS-UGA/trnS-GGA* | LSC | trn |
| 25 | 37295 | F | 67748 | *trnfM-CAU/trnP-UGG* | LSC | trn |
| 21 | 37298 | F | 67752 | *trnfM-CAU/trnP-UGG* | LSC | trn |
| 28 | 39256 | F | 41482 | *psaB/psaA* | LSC | CDS |
| 30 | 39301 | F | 41525 | *psaB/psaA* | LSC | CDS |
| 21 | 42534 | P | 68793 | *psaA-ycf3/psaJ-rpl33* | LSC | IGS |
| 28 | 43767 | F | 68005 | *ycf3/trnP-UGG-psaJ* | LSC | IGS/Intron |
| 27 | 44448 | F | 99584 | *ycf3/rps12-trnV-GAC* | LSC/IR | IGS/Intron |
| 27 | 44448 | F | 122361 | *ycf3/ndhA* | LSC/SSC | Intron |
| 23 | 44452 | F | 99588 | *ycf3/rps12-trnV-GAC* | LSC/IR | IGS/Intron |
| 23 | 44452 | F | 122365 | *ycf3/ndhA* | LSC/SSC | Intron |
| 20 | 48870 | P | 52988 | *trnL-UAA-trnF-GAA/trnV-UAC* | LSC | IGS/trn |
| 19 | 48870 | F | 104227 | *trnL-UAA-trnF-GAA/trnA-UGC* | LSC/IR | IGS/trn |
| 25 | 49053 | P | 49081 | *trnF-GAA-ndhJ* | LSC | IGS |
| 20 | 50949 | R | 50949 | *ndhC* | LSC | CDS |
| 20 | 52989 | P | 104226 | *trnV-UAC/trnA-UGC* | LSC/IR | trn |
| 22 | 53398 | P | 53429 | *trnM-CAU-atpE* | LSC | IGS |
| 27 | 60284 | F | 68525 | *accD-psaI/psaJ-rpl33* | LSC | IGS |
| 32 | 60350 | P | 60350 | *accD-psaI* | LSC | IGS |
| 27 | 62629 | P | 71289 | *cemA/rps12-clpP* | LSC | CDS/IGS |
| 20 | 62638 | P | 71289 | *cemA/rps12-clpP* | LSC | CDS/IGS |
| 19 | 68011 | P | 128521 | *trnP-UGG-psaJ/ycf1* | LSC/SSC | CDS/IGS |
| 29 | 68515 | P | 69154 | *psaJ-rpl33/rpl33-rps18* | LSC | IGS |
| 31 | 69160 | P | 69171 | *rpl33-rps18* | LSC | IGS |
| 27 | 73532 | F | 100131 | *clpP-psbB/rps12-trnV-GAC* | LSC/IR | IGS |
| 23 | 73545 | F | 100126 | *clpP-psbB/rps12-trnV-GAC* | LSC/IR | IGS |
| 19 | 75744 | P | 75769 | *psbT-psbN* | LSC | IGS |
| 29 | 92247 | F | 92283 | *ycf2* | IR | CDS |
| 34 | 92256 | F | 92292 | *ycf2* | IR | CDS |
| 31 | 92277 | F | 92295 | *ycf2* | IR | CDS |
| 25 | 94946 | P | 94983 | *ycf2-trnL-CAA* | IR | IGS |
| 20 | 95090 | P | 116148 | *trnL-CAA/trnL-UAG* | SSC/IR | trn |
| 40 | 99572 | F | 122349 | *rps12-trnV-GAC/ndhA* | SSC/IR | IGS/Intron |
| 22 | 99589 | F | 122367 | *rps12-trnV-GAC/ndhA* | SSC/IR | IGS/Intron |
| 34 | 108337 | F | 108369 | *rrn4.5-rrn5* | IR | IGS |
| 43 | 108670 | P | 108670 | *rrn5-trnR-ACG* | IR | IGS |
| 20 | 112774 | P | 112774 | *ndhF* | SSC | CDS |
| 25 | 115131 | P | 128517 | *rpl32-trnL-UAG/ycf1* | SSC | CDS/IGS |
| 23 | 115134 | P | 128517 | *rpl32-trnL-UAG/ycf1* | SSC | CDS/IGS |
| 27 | 115919 | F | 115944 | *rpl32-trnL-UAG* | SSC | IGS |
| 50 | 119053 | P | 119053 | *ndhD-psaC* | SSC | IGS |

**Supplementary Table 5.** *S. sesuvioides* and 28 chosen NCBI genomes for phylogenetic tree analysis.

| **Order** | **Family** | **Species** | **Gene bank accession number** |
| --- | --- | --- | --- |
| Caryophyllales | Aizoaceae | ***Sesuvium sesuvioides*** | MW539047 |
|  |  | *Sesuvium portulacastrum* | MK330004 |
|  |  | *Mesembryanthemum crystallinum* | KM016695 |
|  |  | *Mesembryanthemum cordifolium* | MK397873 |
|  |  | *Tetragonia tetragonoides* | MF975369 |
|  | Achatocarpaceae | *Phaulothamnus spinescens* | MH286322 |
|  | Amaranthaceae | *Amaranthus blitum* | MW255966 |
|  | Agdestidaceae | *Agdestis clematidea* | MH286339 |
|  | Basellaceae | *Anredera cordifolia* | MW582603 |
|  | Cactaceae | *Opuntia quimilo* | MN114084 |
|  | Caryophyllaceae | *Agrostemma githago* | KF527884 |
|  | Chenopodiaceae | *Chenopodium acuminatum* | MW057780 |
|  | Droseraceae | *Drosera regia* | KY679199 |
|  | Drosophyllaceae | *Drosophyllum lusitanicum* | MH286319 |
|  | Molluginaceae | *Glinus dahomensis* | MH286335 |
|  | Montiaceae | *Cistanthe longiscapa* | KX928992 |
|  | Nepenthaceae | *Nepenthes khasiana* | MH923233 |
|  | Nyctaginaceae | *Nyctaginia capitata* | MH286318 |
|  | Petiveriaceae | *Petiveria alliacea* | MH286334 |
|  | Phytolaccaceae | *Phytolacca acinosa* | OM403715 |
|  | Plumbaginaceae | *Plumbago auriculata* | MH286308 |
|  | Polygonaceae | *Polygonum aviculare* | MZ748474 |
|  | Portulacaceae | *Portulaca gilliesii* | OM691697 |
|  | Sarcobataceae | *Sarcobatus vermiculatus* | MH286338 |
|  | Stegnospermataceae | *Stegnosperma halimifolium* | MH286336 |
|  | Talinaceae | *Talinum paniculatum* | MG710385 |
|  | Tamaricaceae | *Tamarix chinensis* | MN229512 |
| OUTGROUP Gentianales | Apocynaceae | *Asclepias nivea* | KF539844 |
|  |  | *Asclepias syriaca* | KF386166 |

**Supplementary Table 6:** The non-synonymous (Ka) and synonymous (Ks) rates of substitution, as well as the Ka/Ks ratio in *S. sesuvioides*.

| **GENES** | **Ks** | **Ka** | **Ka/Ks** | **Species that Pairwise aligned with *S. sesuvioides*** | **GENES** | **Ks** | **Ka** | **Ka/Ks** |
| --- | --- | --- | --- | --- | --- | --- | --- | --- |
| ***accD*** | 0.0129 | 0.0058 | 0.45 | *S. portulacastrum* | ***psbH*** | 0 | 0 | 0.00 |
|  | 0.1201 | 0.049 | 0.41 | *M. crystallinum* |  | 0.0533 | 0.0056 | 0.11 |
|  | 0.1222 | 0.0484 | 0.40 | *M. cordifolium* |  | 0.0533 | 0.0056 | 0.11 |
|  | 0.1447 | 0.0585 | 0.40 | *T. tetragonoides* |  | 0.1386 | 0.0123 | 0.09 |
| ***atpA*** | 0.0271 | 0.0017 | 0.06 | *S. portulacastrum* | ***psbI*** | 0.0385 | 0 | 0.00 |
|  | 0.1565 | 0.0035 | 0.02 | *M. crystallinum* |  | 0.1219 | 0 | 0.00 |
|  | 0.1598 | 0.0044 | 0.03 | *M. cordifolium* |  | 0.1219 | 0 | 0.00 |
|  | 0.1482 | 0.0101 | 0.07 | *T. tetragonoides* |  | 0.1219 | 0 | 0.00 |
| ***atpB*** | 0.0162 | 0.0045 | 0.28 | *S. portulacastrum* | ***psbJ*** | 0 | 0 | 0.00 |
|  | 0.1282 | 0.0054 | 0.04 | *M. crystallinum* |  | 0.0309 | 0 | 0.00 |
|  | 0.1252 | 0.0054 | 0.04 | *M. cordifolium* |  | 0 | 0 | 0.00 |
|  | 0.1138 | 0.0094 | 0.08 | *T. tetragonoides* |  | 0.0309 | 0 | 0.00 |
| ***atpE*** | 0 | 0 | 0.00 | *S. portulacastrum* | ***psbK*** | 0.0254 | 0 | 0.00 |
|  | 0.1809 | 0.0066 | 0.04 | *M. crystallinum* |  | 0.1073 | 0 | 0.00 |
|  | 0.1809 | 0.0066 | 0.04 | *M. cordifolium* |  | 0.1367 | 0 | 0.00 |
|  | 0.1935 | 0.0033 | 0.02 | *T. tetragonoides* |  | 0.078 | 0.0148 | 0.19 |
| ***atpF*** | 0.0254 | 0 | 0.00 | *S. portulacastrum* | ***psbL*** | 0.0411 | 0 | 0.00 |
|  | 0.1087 | 0.0071 | 0.07 | *M. crystallinum* |  | 0.0403 | 0.0114 | 0.28 |
|  | 0.1174 | 0.0093 | 0.08 | *M. cordifolium* |  | 0.0403 | 0.0114 | 0.28 |
|  | 0.1361 | 0.007 | 0.05 | *T. tetragonoides* |  | 0.0403 | 0.0114 | 0.28 |
| ***atpH*** | 0 | 0 | 0.00 | *S. portulacastrum* | ***psbM*** | 0.0417 | 0 | 0.00 |
|  | 0.0978 | 0 | 0.00 | *M. crystallinum* |  | 0 | 0.013 | 0.00 |
|  | 0.0978 | 0 | 0.00 | *M. cordifolium* |  | 0 | 0.013 | 0.00 |
|  | 0.047 | 0.0057 | 0.12 | *T. tetragonoides* |  | 0 | 0.013 | 0.00 |
| ***atpI*** | 0.011 | 0 | 0.00 | *S. portulacastrum* | ***psbN*** | 0.0321 | 0 | 0.00 |
|  | 0.1384 | 0.0072 | 0.05 | *M. crystallinum* |  | 0.0656 | 0 | 0.00 |
|  | 0.1384 | 0.0072 | 0.05 | *M. cordifolium* |  | 0.0656 | 0 | 0.00 |
|  | 0.1517 | 0.0072 | 0.05 | *T. tetragonoides* |  | 0.1019 | 0 | 0.00 |
| ***ccsA*** | 0.0529 | 0.0066 | 0.12 | *S. portulacastrum* | ***psbT*** | 0 | 0 | 0.00 |
|  | 0.1519 | 0.0335 | 0.22 | *M. crystallinum* |  | 0.0411 | 0.0264 | 0.64 |
|  | 0.1632 | 0.0335 | 0.21 | *M. cordifolium* |  | 0.0411 | 0.0264 | 0.64 |
|  | 0.1612 | 0.0327 | 0.20 | *T. tetragonoides* |  | 0.0877 | 0.0579 | 0.66 |
| ***cemA*** | 0.0138 | 0.0019 | 0.14 | *S. portulacastrum* | ***psbZ*** | 0.0445 | 0 | 0.00 |
|  | 0.0647 | 0.0074 | 0.11 | *M. crystallinum* |  | 0.0672 | 0 | 0.00 |
|  | 0.0646 | 0.0112 | 0.17 | *M. cordifolium* |  | 0.0672 | 0 | 0.00 |
|  | 0.0957 | 0.0149 | 0.16 | *T. tetragonoides* |  | 0.0217 | 0 | 0.00 |
| ***clpP*** | 0.0219 | 0 | 0.00 | *S. portulacastrum* | ***rbcL*** | 0.0145 | 0.0074 | 0.51 |
|  | 0.052 | 0 | 0.00 | *M. crystallinum* |  | 0.1314 | 0.0157 | 0.12 |
|  | 0.0445 | 0 | 0.00 | *M. cordifolium* |  | 0.1279 | 0.0139 | 0.11 |
|  | 0.0602 | 0 | 0.00 | *T. tetragonoides* |  | 0.1145 | 0.0204 | 0.18 |
| ***matK*** | 0.036 | 0.0094 | 0.26 | *S. portulacastrum* | ***rpl2*** | 0.0049 | 0 | 0.00 |
|  | 0.1485 | 0.0513 | 0.35 | *M. crystallinum* |  | 0.0247 | 0.0049 | 0.20 |
|  | 0.1385 | 0.0492 | 0.36 | *M. cordifolium* |  | 0.0298 | 0.0065 | 0.22 |
|  | 0.2045 | 0.0468 | 0.23 | *T. tetragonoides* |  | 0.04 | 0.0049 | 0.12 |
| ***ndhA*** | 0.0262 | 0.0025 | 0.10 | *S. portulacastrum* | ***rpl14*** | 0.0232 | 0.0036 | 0.16 |
|  | 0.1243 | 0.0186 | 0.15 | *M. crystallinum* |  | 0.0349 | 0.0036 | 0.10 |
|  | 0.1372 | 0.0173 | 0.13 | *M. cordifolium* |  | 0.0349 | 0.0036 | 0.10 |
|  | 0.1298 | 0.0155 | 0.12 | *T. tetragonoides* |  | 0.0838 | 0.0036 | 0.04 |
| ***ndhB*** | 0.0081 | 0 | 0.00 | *S. portulacastrum* | ***rpl16*** | 0.0097 | 0 | 0.00 |
|  | 0.0163 | 0.0035 | 0.21 | *M. crystallinum* |  | 0.12 | 0.004 | 0.03 |
|  | 0.0163 | 0.0035 | 0.21 | *M. cordifolium* |  | 0.1146 | 0.0033 | 0.03 |
|  | 0.019 | 0.0026 | 0.14 | *T. tetragonoides* |  | 0.1599 | 0.0067 | 0.04 |
| ***ndhC*** | 0 | 0 | 0.00 | *S. portulacastrum* | ***rpl20*** | 0.0111 | 0.0069 | 0.62 |
|  | 0.0749 | 0.0036 | 0.05 | *M. crystallinum* |  | 0.0699 | 0.0242 | 0.35 |
|  | 0.0883 | 0.0073 | 0.08 | *M. cordifolium* |  | 0.0699 | 0.0242 | 0.35 |
|  | 0.0751 | 0 | 0.00 | *T. tetragonoides* |  | 0.1071 | 0.0277 | 0.26 |
| ***ndhD*** | 0.038 | 0.0045 | 0.12 | *S. portulacastrum* | ***rpl22*** | 0.085 | 0.0191 | 0.22 |
|  | 0.1786 | 0.0162 | 0.09 | *M. crystallinum* |  | 0.2595 | 0.3512 | 1.35 |
|  | 0.1749 | 0.0153 | 0.09 | *M. cordifolium* |  | 0.2518 | 0.3268 | 1.30 |
|  | 0.2041 | 0.0153 | 0.07 | *T. tetragonoides* |  | 0.4487 | 0.266 | 0.59 |
| ***ndhE*** | 0.0573 | 0 | 0.00 | *S. portulacastrum* | ***rpl23*** | 0 | 0 | 0.00 |
|  | 0.1686 | 0.022 | 0.13 | *M. crystallinum* |  | 0.0157 | 0 | 0.00 |
|  | 0.1686 | 0.022 | 0.13 | *M. cordifolium* |  | 0.0157 | 0 | 0.00 |
|  | 0.2251 | 0.0043 | 0.02 | *T. tetragonoides* |  | N/A | N/A | 0.00 |
| ***ndhF*** | 0.028 | 0.0111 | 0.40 | *S. portulacastrum* | ***rpl32*** | 0.0268 | 0.0395 | 1.47 |
|  | 0.1722 | 0.0361 | 0.21 | *M. crystallinum* |  | 0.1941 | 0.1539 | 0.79 |
|  | 0.177 | 0.0399 | 0.23 | *M. cordifolium* |  | 0.2136 | 0.1674 | 0.78 |
|  | 0.1995 | 0.0385 | 0.19 | *T. tetragonoides* |  | 0.0828 | 0.1404 | 1.70 |
| ***ndhG*** | 0.0078 | 0.0025 | 0.32 | *S. portulacastrum* | ***rpl33*** | 0.0224 | 0.0066 | 0.29 |
|  | 0.1081 | 0.0101 | 0.09 | *M. crystallinum* |  | 0.1193 | 0.0267 | 0.22 |
|  | 0.1262 | 0.0101 | 0.08 | *M. cordifolium* |  | 0.1193 | 0.0267 | 0.22 |
|  | 0.0991 | 0.0101 | 0.10 | *T. tetragonoides* |  | 0.0939 | 0.0335 | 0.36 |
| ***ndhH*** | 0.0115 | 0.0044 | 0.38 | *S. portulacastrum* | ***rpl36*** | 0.0775 | 0 | 0.00 |
|  | 0.1152 | 0.0088 | 0.08 | *M. crystallinum* |  | 0.0775 | 0 | 0.00 |
|  | 0.1109 | 0.0088 | 0.08 | *M. cordifolium* |  | 0.1195 | 0 | 0.00 |
|  | 0.149 | 0.0082 | 0.06 | *T. tetragonoides* |  | 0.1634 | 0 | 0.00 |
| ***ndhI*** | 0.0084 | 0 | 0.00 | *S. portulacastrum* | ***rpoA*** | 0.0223 | 0.0025 | 0.11 |
|  | 0.2081 | 0.0091 | 0.04 | *M. crystallinum* |  | 0.1366 | 0.0242 | 0.18 |
|  | 0.1757 | 0.0143 | 0.08 | *M. cordifolium* |  | 0.1475 | 0.0256 | 0.17 |
|  | 0.1804 | 0.0155 | 0.09 | *T. tetragonoides* |  | 0.1249 | 0.0278 | 0.22 |
| ***ndhJ*** | 0.0185 | 0 | 0.00 | *S. portulacastrum* | ***rpoB*** | 0.0174 | 0.002 | 0.11 |
|  | 0.1181 | 0.0083 | 0.07 | *M. crystallinum* |  | 0.1003 | 0.0144 | 0.14 |
|  | 0.1288 | 0.0083 | 0.06 | *M. cordifolium* |  | 0.1018 | 0.0144 | 0.14 |
|  | 0.1409 | 0.0138 | 0.10 | *T. tetragonoides* |  | 0.1101 | 0.0146 | 0.13 |
| ***ndhK*** | 0.0247 | 0.0039 | 0.16 | *S. portulacastrum* | ***rpoC1*** | 0.0149 | 0.0013 | 0.09 |
|  | 0.0891 | 0.0203 | 0.23 | *M. crystallinum* |  | 0.0872 | 0.0077 | 0.09 |
|  | 0.0891 | 0.0203 | 0.23 | *M. cordifolium* |  | 0.0871 | 0.0077 | 0.09 |
|  | 0.1385 | 0.0264 | 0.19 | *T. tetragonoides* |  | 0.1 | 0.0084 | 0.08 |
| ***petA*** | 0 | 0.0041 | 0.00 | *S. portulacastrum* | ***rpoC2*** | 0.0235 | 0.0054 | 0.23 |
|  | 0.1086 | 0.0069 | 0.06 | *M. crystallinum* |  | 0.1183 | 0.0247 | 0.21 |
|  | 0.0885 | 0.0069 | 0.08 | *M. cordifolium* |  | 0.1218 | 0.0261 | 0.21 |
|  | 0.124 | 0.0055 | 0.04 | *T. tetragonoides* |  | 0.1342 | 0.0277 | 0.21 |
| ***petB*** | 0.0321 | 0.0021 | 0.07 | *S. portulacastrum* | ***rps2*** | 0.0314 | 0.0037 | 0.12 |
|  | 0.1381 | 0.0062 | 0.04 | *M. crystallinum* |  | 0.1271 | 0 | 0.00 |
|  | 0.1302 | 0.0062 | 0.05 | *M. cordifolium* |  | 0.1199 | 0.0018 | 0.02 |
|  | 0.1768 | 0.0062 | 0.04 | *T. tetragonoides* |  | 0.1056 | 0.0018 | 0.02 |
| ***petD*** | 0.017 | 0.0028 | 0.16 | *S. portulacastrum* | ***rps3*** | 0.0343 | 0 | 0.00 |
|  | 0.0998 | 0.0056 | 0.06 | *M. crystallinum* |  | 0.1613 | 0.0089 | 0.06 |
|  | 0.1085 | 0.0028 | 0.03 | *M. cordifolium* |  | 0.1529 | 0.011 | 0.07 |
|  | 0.0893 | 0 | 0.00 | *T. tetragonoides* |  | 0.1447 | 0.013 | 0.09 |
| ***petG*** | 0 | 0 | 0.00 | *S. portulacastrum* | ***rps4*** | 0.0279 | 0 | 0.00 |
|  | 0.1164 | 0 | 0.00 | *M. crystallinum* |  | 0.1266 | 0.011 | 0.09 |
|  | 0.1164 | 0 | 0.00 | *M. cordifolium* |  | 0.1108 | 0.011 | 0.10 |
|  | 0.1574 | 0.0122 | 0.08 | *T. tetragonoides* |  | 0.0947 | 0.011 | 0.12 |
| ***petL*** | 0 | 0 | 0.00 | *S. portulacastrum* | ***rps7*** | 0 | 0 | 0.00 |
|  | 0.0817 | 0.015 | 0.18 | *M. crystallinum* |  | 0 | 0 | 0.00 |
|  | 0.0828 | 0.0302 | 0.36 | *M. cordifolium* |  | 0.0178 | 0.0057 | 0.32 |
|  | 0.128 | 0.015 | 0.12 | *T. tetragonoides* |  | 0 | 0 | 0.00 |
| ***petN*** | 0.0488 | 0 | 0.00 | *S. portulacastrum* | ***rps8*** | 0.0101 | 0 | 0.00 |
|  | 0.1585 | 0 | 0.00 | *M. crystallinum* |  | 0.0732 | 0.0168 | 0.23 |
|  | 0.1585 | 0 | 0.00 | *M. cordifolium* |  | 0.0732 | 0.0168 | 0.23 |
|  | 0.1585 | 0 | 0.00 | *T. tetragonoides* |  | 0.085 | 0.0133 | 0.16 |
| ***psaA*** | 0.027 | 0.0006 | 0.02 | *S. portulacastrum* | ***rps11*** | 0.0487 | 0 | 0.00 |
|  | 0.1052 | 0.0012 | 0.01 | *M. crystallinum* |  | 0.1227 | 0.0098 | 0.08 |
|  | 0.1061 | 0.0024 | 0.02 | *M. cordifolium* |  | 0.1453 | 0.0098 | 0.07 |
|  | 0.1165 | 0.0023 | 0.02 | *T. tetragonoides* |  | 0.1811 | 0.0033 | 0.02 |
| ***psaB*** | 0.014 | 0.0006 | 0.04 | *S. portulacastrum* | ***rps12*** | 0.021 | 0.0037 | 0.18 |
|  | 0.1294 | 0.0018 | 0.01 | *M. crystallinum* |  | 0.0498 | 0.0333 | 0.67 |
|  | 0.1294 | 0.0024 | 0.02 | *M. cordifolium* |  | 0.0209 | 0.0074 | 0.35 |
|  | 0.1509 | 0.003 | 0.02 | *T. tetragonoides* |  | 0.0316 | 0.0074 | 0.23 |
| ***psaC*** | 0 | 0 | 0.00 | *S. portulacastrum* | ***rps14*** | 0 | 0.0043 | 0.00 |
|  | 0.1564 | 0 | 0.00 | *M. crystallinum* |  | 0.0463 | 0.0086 | 0.19 |
|  | 0.1564 | 0 | 0.00 | *M. cordifolium* |  | 0.0463 | 0.0086 | 0.19 |
|  | 0.2484 | 0 | 0.00 | *T. tetragonoides* |  | 0.0619 | 0.0174 | 0.28 |
| ***psaI*** | 0 | 0 | 0.00 | *S. portulacastrum* | ***rps16*** | 0.051 | 0 | 0.00 |
|  | 0.0397 | 0.0247 | 0.62 | *M. crystallinum* |  | 0.1282 | 0.0113 | 0.09 |
|  | 0.0397 | 0.0374 | 0.94 | *M. cordifolium* |  | 0.1247 | 0.0056 | 0.04 |
|  | 0 | 0.0632 | 0.00 | *T. tetragonoides* |  | 0.1244 | 0.028 | 0.23 |
| ***psaJ*** | 0.0318 | 0 | 0.00 | *S. portulacastrum* | ***rps18*** | 0.0439 | 0 | 0.00 |
|  | 0.1742 | 0 | 0.00 | *M. crystallinum* |  | 0.0441 | 0.0169 | 0.38 |
|  | 0.1742 | 0.0101 | 0.06 | *M. cordifolium* |  | 0.0441 | 0.0169 | 0.38 |
|  | 0.1352 | 0.0101 | 0.07 | *T. tetragonoides* |  | 0.0297 | 0.0216 | 0.73 |
| ***psbA*** | 0.0244 | 0 | 0.00 | *S. portulacastrum* | ***rps19*** | 0.0825 | 0.0047 | 0.06 |
|  | 0.1027 | 0 | 0.00 | *M. crystallinum* |  | 0.2369 | 0.0143 | 0.06 |
|  | 0.1027 | 0 | 0.00 | *M. cordifolium* |  | 0.2145 | 0.0143 | 0.07 |
|  | 0.0625 | 0 | 0.00 | *T. tetragonoides* |  | 0.3056 | 0.0047 | 0.02 |
| ***psbB*** | 0.0167 | 0 | 0.00 | *S. portulacastrum* | ***ycf1*** | 0.0254 | 0.016 | 0.63 |
|  | 0.1446 | 0.0035 | 0.02 | *M. crystallinum* |  | 0.1428 | 0.0894 | 0.63 |
|  | 0.1479 | 0.0026 | 0.02 | *M. cordifolium* |  | 0.1454 | 0.0888 | 0.61 |
|  | 0.158 | 0.0069 | 0.04 | *T. tetragonoides* |  | 0.0793 | 0.031 | 0.39 |
| ***psbC*** | 0.0057 | 0.0009 | 0.16 | *S. portulacastrum* | ***ycf2*** | 0.0013 | 0.0013 | 1.00 |
|  | 0.1078 | 0.0009 | 0.01 | *M. crystallinum* |  | 0.0201 | 0.0123 | 0.61 |
|  | 0.1011 | 0.0009 | 0.01 | *M. cordifolium* |  | 0.0175 | 0.0118 | 0.67 |
|  | 0.1212 | 0.0028 | 0.02 | *T. tetragonoides* |  | 0.0193 | 0.0179 | 0.93 |
| ***psbD*** | 0.0164 | 0 | 0.00 | *S. portulacastrum* | ***ycf3*** | 0 | 0.0026 | 0.00 |
|  | 0.0946 | 0.0037 | 0.04 | *M. crystallinum* |  | 0.0809 | 0.0052 | 0.06 |
|  | 0.0992 | 0.0037 | 0.04 | *M. cordifolium* |  | 0.0809 | 0.0052 | 0.06 |
|  | 0.0993 | 0.0037 | 0.04 | *T. tetragonoides* |  | 0.1003 | 0.0026 | 0.03 |
| ***psbE*** | 0.0174 | 0 | 0.00 | *S. portulacastrum* | ***ycf4*** | 0.0155 | 0 | 0.00 |
|  | 0.1522 | 0 | 0.00 | *M. crystallinum* |  | 0.1075 | 0.0071 | 0.07 |
|  | 0.1522 | 0 | 0.00 | *M. cordifolium* |  | 0.1075 | 0.0071 | 0.07 |
|  | 0.173 | 0.0053 | 0.03 | *T. tetragonoides* |  | 0.1444 | 0.0119 | 0.08 |
| ***psbF*** | 0 | 0 | 0.00 | *S. portulacastrum* |  | | | |
|  | 0.0332 | 0.0117 | 0.35 | *M. crystallinum* |  |  |  |  |
|  | 0 | 0.0117 | 0.00 | *M. cordifolium* |  |  |  |  |
|  | 0 | 0.0354 | 0.00 | *T. tetragonoides* |  |  |  |  |

**Supplementary Table 7: S**ingle nucleotide polymorphisms (SNPs) in LSC, IR, and SSC regions of *S. sesuvioides* by making pairwise alignment with four Aizoaceae other species (*S. portulacastrum, M. crystallinum, M. cordifolium,* and *T. tetragonoides)*.

| **Region** | ***Species (Sesuvium sesuvioides as reference)*** | **Transition substituations** | | **Transversion substituaions** | | | |
| --- | --- | --- | --- | --- | --- | --- | --- |
|  |  | **A/G** | **C/T** | **A/T** | **A/C** | **C/G** | **G/T** |
| **Substitution Type** |  | **R** | **Y** | **W** | **M** | **S** | **K** |
| Large Single Copy | *S. portulacastrum* | 275 | 273 | 126 | 164 | 62 | 153 |
|  | *M. crystallinum* | 1384 | 1457 | 750 | 615 | 195 | 665 |
|  | *M. cordifolium* | 1390 | 1450 | 746 | 642 | 195 | 652 |
|  | *T. tetragonoides* | 1390 | 1445 | 754 | 677 | 217 | 777 |
| Inverted Repeat | *S. portulacastrum* | 12 | 9 | 6 | 8 | 2 | 10 |
|  | *M. crystallinum* | 77 | 88 | 27 | 37 | 13 | 47 |
|  | *M. cordifolium* | 82 | 86 | 21 | 33 | 8 | 48 |
|  | *T. tetragonoides* | 92 | 90 | 43 | 57 | 24 | 68 |
| Small Single Copy | *S. portulacastrum* | 79 | 99 | 29 | 53 | 22 | 59 |
|  | *M. crystallinum* | 406 | 452 | 223 | 205 | 81 | 234 |
|  | *M. cordifolium* | 403 | 472 | 227 | 204 | 88 | 244 |
|  | *T. tetragonoides* | 377 | 440 | 180 | 184 | 82 | 249 |

**Supplementary Table 8:** Nucleotide Diversity and alignment length in coding, non-coding, and intronic regions among *S. sesuvioides* and four other Aizoaceae species (*S. portulacastrum, M. crystallinum, M. cordifolium,* and *T. tetragonoides*.

| **S.No** | **Region** | **Location** | **Nucleotide Diversity** | **T. No's of Mutations** | **Silent positions** | **Alignment Length** |
| --- | --- | --- | --- | --- | --- | --- |
| 1 | *accD* | CDS | 0.0950 | 140 | 1473 | 1542 |
| 2 | *atpA* | CDS | 0.0564 | 86 | 1524 | 1524 |
| 3 | *atpB* | CDS | 0.0481 | 72 | 1497 | 1497 |
| 4 | *atpE* | CDS | 0.0522 | 21 | 402 | 402 |
| 5 | *atpF* | CDS | 0.0435 | 24 | 552 | 555 |
| 6 | *atpH* | CDS | 0.0285 | 7 | 246 | 246 |
| 7 | *atpI* | CDS | 0.0551 | 41 | 744 | 744 |
| 8 | *ccsA* | CDS | 0.0857 | 83 | 969 | 975 |
| 9 | *clpP* | CDS | 0.0221 | 13 | 588 | 594 |
| 10 | *cemA* | CDS | 0.0391 | 27 | 690 | 702 |
| 11 | *matK* | CDS | 0.1078 | 163 | 1512 | 1518 |
| 12 | *ndhA* | CDS | 0.0678 | 74 | 1092 | 1092 |
| 13 | *ndhB* | CDS | 0.0104 | 16 | 1533 | 1602 |
| 14 | *ndhC* | CDS | 0.0303 | 11 | 363 | 363 |
| 15 | *ndhD* | CDS | 0.0860 | 127 | 1476 | 1503 |
| 16 | *ndhE* | CDS | 0.0817 | 25 | 306 | 306 |
| 17 | *ndhF* | CDS | 0.1012 | 214 | 2115 | 2259 |
| 18 | *ndhG* | CDS | 0.0527 | 28 | 531 | 531 |
| 19 | *ndhH* | CDS | 0.0533 | 63 | 1182 | 1182 |
| 20 | *ndhI* | CDS | 0.0747 | 38 | 509 | 517 |
| 21 | *ndhJ* | CDS | 0.0524 | 25 | 477 | 477 |
| 22 | *ndhK* | CDS | 0.0590 | 40 | 678 | 678 |
| 23 | *petA* | CDS | 0.0457 | 44 | 963 | 966 |
| 24 | *petB* | CDS | 0.0571 | 37 | 648 | 687 |
| 25 | *petD* | CDS | 0.0455 | 22 | 483 | 510 |
| 26 | *psaA* | CDS | 0.0448 | 97 | 2166 | 2253 |
| 27 | *psaB* | CDS | 0.0449 | 99 | 2205 | 2205 |
| 28 | *psaC* | CDS | 0.0488 | 12 | 246 | 246 |
| 29 | *psbA* | CDS | 0.0330 | 35 | 1062 | 1062 |
| 30 | *psbB* | CDS | 0.0557 | 85 | 1527 | 1527 |
| 31 | *psbC* | CDS | 0.0387 | 55 | 1422 | 1422 |
| 32 | *psbD* | CDS | 0.0358 | 38 | 1062 | 1062 |
| 33 | *psbE* | CDS | 0.0397 | 10 | 252 | 252 |
| 34 | *psbH* | CDS | 0.0450 | 10 | 222 | 240 |
| 35 | *rbcL* | CDS | 0.0638 | 92 | 1443 | 1449 |
| 36 | *rpl2* | CDS | 0.0198 | 16 | 810 | 825 |
| 37 | *rpl14* | CDS | 0.0301 | 11 | 366 | 366 |
| 38 | *rpl16* | CDS | 0.0546 | 19 | 348 | 411 |
| 39 | *rpl20* | CDS | 0.0620 | 24 | 387 | 387 |
| 40 | *rpl22* | CDS | 0.4789 | 34 | 71 | 606 |
| 41 | *rpl33* | CDS | 0.0547 | 11 | 201 | 201 |
| 42 | *rpoA* | CDS | 0.0713 | 71 | 996 | 1038 |
| 43 | *rpoB* | CDS | 0.0501 | 161 | 3213 | 3213 |
| 44 | *rpoC1* | CDS | 0.0428 | 87 | 2034 | 2061 |
| 45 | *rpoC2* | CDS | 0.0718 | 295 | 4110 | 4137 |
| 46 | *rps2* | CDS | 0.0408 | 29 | 711 | 711 |
| 47 | *rps3* | CDS | 0.0596 | 39 | 654 | 657 |
| 48 | *rps4* | CDS | 0.0561 | 34 | 606 | 606 |
| 49 | *rps7* | CDS | 0.0085 | 4 | 468 | 468 |
| 50 | *rps8* | CDS | 0.0420 | 17 | 405 | 405 |
| 51 | *rps11* | CDS | 0.0743 | 31 | 417 | 417 |
| 52 | *rps12* | CDS | 0.0476 | 17 | 357 | 372 |
| 53 | *rps14* | CDS | 0.0330 | 10 | 303 | 303 |
| 54 | *rps16* | CDS | 0.0741 | 18 | 243 | 273 |
| 55 | *rps18* | CDS | 0.0392 | 12 | 306 | 312 |
| 56 | *rps19* | CDS | 0.1004 | 28 | 279 | 279 |
| 57 | *ycf1* | CDS | 0.0651 | 109 | 1674 | 5796 |
| 58 | *ycf2* | CDS | 0.0237 | 122 | 5154 | 6885 |
| 59 | *ycf3* | CDS | 0.0276 | 14 | 507 | 510 |
| 60 | *ycf4* | CDS | 0.0559 | 31 | 555 | 555 |
| 61 | *trnH-GUG-psbA* | IGS | 0.3088 | 63 | 204 | 384 |
| 62 | *psbA-trnK-UUU* | IGS | 0.1726 | 34 | 197 | 233 |
| 63 | *trnK-UUU Intron* | Intron | 0.1108 | 272 | 2455 | 2581 |
| 64 | *trnK-UUU-rps16* | IGS | 0.3163 | 99 | 313 | 895 |
| 65 | *rps16 Intron* | Intron | 0.1600 | 124 | 775 | 974 |
| 66 | *rps16-trnQ-UUG* | IGS | 0.2407 | 65 | 270 | 926 |
| 67 | *trnQ-UUG-psbK* | IGS | 0.1239 | 41 | 331 | 375 |
| 68 | *psbK-psbI* | IGS | 0.1241 | 50 | 403 | 490 |
| 69 | *atpF Intron* | Intron | 0.0954 | 67 | 702 | 801 |
| 70 | *atpF-atpH* | IGS | 0.1496 | 54 | 361 | 443 |
| 71 | *atpH-atpI* | IGS | 0.1552 | 97 | 625 | 780 |
| 72 | *atpI-rps2* | IGS | 0.1782 | 36 | 202 | 223 |
| 73 | *rps2-rpoC2* | IGS | 0.0979 | 23 | 235 | 246 |
| 74 | *rpoC2-rpoC1* | IGS | 0.1135 | 21 | 185 | 239 |
| 75 | *rpoC1 Intron* | Intron | 0.0986 | 73 | 740 | 826 |
| 76 | *rpoB-trnC-GCA* | IGS | 0.1920 | 167 | 870 | 1196 |
| 77 | *trnC-GCA-petN* | IGS | 0.2118 | 129 | 609 | 662 |
| 78 | *petN-psbM* | IGS | 0.1247 | 47 | 377 | 1185 |
| 79 | *psbM-trnD-GUC* | IGS | 0.2113 | 131 | 620 | 1290 |
| 80 | *trnD-GUC-trnY-GUA* | IGS | 0.4041 | 78 | 193 | 461 |
| 81 | *trnE-UUC-trnT-GGU* | IGS | 0.3159 | 145 | 459 | 761 |
| 82 | *trnT-GGU-psbD* | IGS | 0.1615 | 178 | 1102 | 1453 |
| 83 | *psbC-trnS-UGA* | IGS | 0.2273 | 45 | 198 | 217 |
| 84 | *trnS-UGA-psbZ* | IGS | 0.1228 | 41 | 334 | 389 |
| 85 | *psaA-ycf3* | IGS | 0.2215 | 144 | 650 | 885 |
| 86 | *ycf3 Intron2* | Intron | 0.0804 | 59 | 734 | 784 |
| 87 | *ycf3 Intron1* | Intron | 0.0768 | 58 | 755 | 813 |
| 88 | *ycf3-trnS-GGA* | IGS | 0.2560 | 117 | 457 | 819 |
| 89 | *trnS-GGA-rps4* | IGS | 0.1356 | 32 | 236 | 358 |
| 90 | *rps4-trnT-UGU* | IGS | 0.2689 | 71 | 264 | 485 |
| 91 | *trnT-UGU-trnL-UAA* | IGS | 0.2177 | 108 | 496 | 814 |
| 92 | *trnL-UAA Intron* | Intron | 0.0860 | 30 | 349 | 553 |
| 93 | *trnL-UAA-trnF-GAA* | IGS | 0.2025 | 66 | 326 | 386 |
| 94 | *trnF-GAA-ndhJ* | IGS | 0.2615 | 74 | 283 | 723 |
| 95 | *ndhC-trnV-UAC* | IGS | 0.2549 | 156 | 612 | 1300 |
| 96 | *trnV-UAC Intron* | Intron | 0.0667 | 39 | 585 | 625 |
| 97 | *trnV-UAC-trnM-CAU* | IGS | 0.1012 | 17 | 168 | 202 |
| 98 | *trnM-CAU-atpE* | IGS | 0.3119 | 68 | 218 | 295 |
| 99 | *atpB-rbcL* | IGS | 0.0965 | 71 | 736 | 870 |
| 100 | *rbcL-accD* | IGS | 0.1465 | 93 | 635 | 761 |
| 101 | *accD-psaI* | IGS | 0.2237 | 98 | 438 | 673 |
| 102 | *psaI-ycf4* | IGS | 0.1164 | 34 | 292 | 318 |
| 103 | *ycf4-cemA* | IGS | 0.2185 | 104 | 476 | 960 |
| 104 | *cemA-petA* | IGS | 0.1660 | 40 | 241 | 295 |
| 105 | *petA-psbJ* | IGS | 0.1629 | 143 | 878 | 1027 |
| 106 | *psbE-petL* | IGS | 0.1517 | 122 | 804 | 1204 |
| 107 | *trnP-UGG-psaJ* | IGS | 0.2355 | 81 | 344 | 459 |
| 108 | *psaJ-rpl33* | IGS | 0.3088 | 134 | 434 | 524 |
| 109 | *rpl33-rps18* | IGS | 0.2426 | 49 | 202 | 295 |
| 110 | *rps18-rpl20* | IGS | 0.1630 | 37 | 227 | 318 |
| 111 | *rpl20-rps12* | IGS | 0.0853 | 62 | 727 | 813 |
| 112 | *clpP Intron2* | Intron | 0.1160 | 68 | 586 | 656 |
| 113 | *clpP Intron1* | Intron | 0.1405 | 117 | 833 | 982 |
| 114 | *clpP-psbB* | IGS | 0.1030 | 44 | 427 | 489 |
| 115 | *psbB-psbT* | IGS | 0.2023 | 35 | 173 | 222 |
| 116 | *psbH-petB* | IGS | 0.0873 | 11 | 126 | 858 |
| 117 | *rpl16-rps3* | IGS | 0.1477 | 22 | 149 | 1231 |
| 118 | *trnL-CAA-ndhB* | IGS | 0.0283 | 16 | 565 | 588 |
| 119 | *ndhB Intron* | Intron | 0.0210 | 14 | 668 | 806 |
| 120 | *ndhB-rps7* | IGS | 0.0339 | 4 | 118 | 315 |
| 121 | *trnI-GAU Intron* | Intron | 0.0075 | 7 | 930 | 948 |
| 122 | *trnA-UGC Intron* | Intron | 0.0024 | 2 | 818 | 825 |
| 123 | *trnR-ACG-trnN-GUU* | IGS | 0.1287 | 66 | 513 | 621 |
| 124 | *ndhF-rpl32* | IGS | 0.2609 | 102 | 391 | 1268 |
| 125 | *rpl32-trnL-UAG* | IGS | 0.3184 | 178 | 559 | 1288 |
| 126 | *ccsA-ndhD* | IGS | 0.2021 | 38 | 188 | 235 |
| 127 | *psaC-ndhE* | IGS | 0.2114 | 63 | 298 | 381 |
| 128 | *ndhE-ndhG* | IGS | 0.2420 | 53 | 219 | 254 |
| 129 | *ndhG-ndhI* | IGS | 0.2473 | 69 | 279 | 369 |
| 130 | *ndhA Intron* | Intron | 0.1491 | 149 | 999 | 1135 |
